# Cysteine Redox State Governs the Condensation Pathway of Hendra Virus W Protein and Differentially Impacts Type I IFN and NF‐κB Signaling

**DOI:** 10.1002/advs.202523465

**Published:** 2026-07-17

**Authors:** Frank Gondelaud, Alexandre Lalande, Giulia Pesce, Auriane Bouchet, Carole Meunier, Christophe Bignon, Denis Ptchelkine, Yu Gu, Eva Ogire, Pierre‐Yves Lozach, Denis Gerlier, Cyrille Mathieu, Sonia Longhi

**Affiliations:** ^1^ Laboratoire Architecture Et Fonction des Macromolécules Biologiques (AFMB) UMR 7257 Aix Marseille University and Centre National De La Recherche Scientifique (CNRS) Marseille France; ^2^ CIRI Centre International de Recherche en Infectiologie Univ Lyon Inserm U1111 CNRS UMR5308 Université Claude Bernard Lyon 1 Ecole Normale Supérieure de Lyon Lyon France; ^3^ IVPC UMR754 INRAE Université Claude Bernard Lyon 1 EPHE Université PSL Lyon France

**Keywords:** cysteines, fibrils, henipaviruses, innate immune response, intrinsically disordered proteins, reactive oxygen species

## Abstract

The Hendra and Nipah viruses (HeV and NiV) are zoonotic biosafety level‐4 pathogens belonging to the *Paramyxoviridae* family. We previously showed that their W protein, a key player in the evasion of the host antiviral response, forms highly flexible, curved fibrils in vitro. Here, we show that the cysteine oxidation state acts as a molecular switch controlling the formation of either amorphous aggregates or flexible fibrils, and that residues 2 to 29 are essential for fibrillation. We also uncover that the HeV W protein (W^HeV^) can also self‐assemble in cellula. W^HeV^ forms distinct types of nuclear condensates that exhibit different dependencies on the cysteine redox state. While deletion of residues 2–29 prevents formation of nuclear filaments, cysteine‐to‐serine substitution mainly impairs the formation of non‐filamentous condensates. Both infection and W^HeV^ ectopic expression trigger oxidative stress, presumably favorable to W^HeV^ condensation. Finally, we show that impaired ability to form redox‐sensitive, non‐filamentous condensates is associated with a reduced W ability to inhibit the NF‐κB pathway, while it conversely enhances W ability to repress the interferon response pathway by enhancing W binding to STAT1.

## Graphical abstract

The cysteine redox state of the Hendra virus W (W^HeV^) protein controls assembly into fibrils or amorphous aggregates, with residues 2–29 required for fibrillation. In cells, W^HeV^ forms different types of nuclear condensates. Impaired ability to form redox‐sensitive, non‐filamentous condensates reduces W^HeV^ inhibition of the NF‐κB pathway, while it conversely enhances its ability to repress the interferon response.

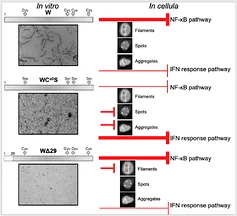

## 1 Introduction

Hendra (HeV) and Nipah viruses (NiV) are members of the *Henipavirus* genus, within the *Paramyxoviridae* family of the *Mononegavirales* order. They are zoonotic viruses responsible for severe encephalitis and respiratory illness with a high case fatality, and as such, they are considered high‐priority targets by the WHO. Since the identification of HeV and NiV in humans and fruit‐bats from the *Pteropus* genus [1, 2], new species have been described, including the Cedar virus identified in bats [3], the Langya virus identified in patients, bats, and shrews [4, 5], and two novel species in shrews [6]. Transmission from bats to humans occurs through the consumption of raw date palm sap (NiV) or from close contact with infected livestock, particularly horses (HeV) and pigs (NiV) [7]. Interhuman transmissions also occurred in the case of NiV Bangladesh strain [8]. Henipavirus outbreaks are unpredictable but occur almost every year with a high case fatality rate (70%–100%) while no antiviral therapeutic or vaccine is currently available [2, 9]. For these reasons, HeV and NiV are considered biosafety level‐4 pathogens.

Henipaviruses, like all paramyxoviruses, are enveloped viruses containing a non‐segmented single‐stranded RNA genome of negative polarity (‐ssRNA) tightly associated with the nucleoprotein (N). The encapsidated RNA is the template of the viral RNA‐dependent RNA polymerase (L) that, in association with the phosphoprotein (P), ensures the transcription and the replication of the viral genome [2]. Beyond the P protein, the P gene also drives the synthesis of the V, W, and C proteins. The V and W proteins are produced through a co‐transcriptional editing mechanism involving the addition of one (V) or two (W) non‐templated G nucleotides at the editing site of the P messenger [1]. As a result, the P, V, and W proteins share a common N‐terminal domain (NTD), and have unique and distinct C‐terminal domains (CTD). The W proteins of HeV and NiV contain a nuclear localization sequence (NLS) in their unique CTD, leading to their nuclear accumulation in infected cells. Nuclear importation relies on binding to importins α3 and α4 and, to a lower extent, to importin α1 and α7 [10]. W, along with P and V, also contains a nuclear export signal (NES) in its NTD, ensuring its export to the cytoplasm of infected cells via a Crm‐1‐dependent nuclear export [11, 12]. Since W is almost found in the nucleus of infected cells, the NLS overcomes the NES [13, 14].

The V and W proteins are virulence factors implicated in the inhibition of the antiviral response by binding several key cellular proteins [2, 13, 15, 16, 17]. The lack of the V and W proteins in the non‐pathogenic Cedar virus due to the absence of the conventional RNA editing site in P gene, concomitantly with the absence of pathogenicity associated with this virus in humans, further supports the critical role of these proteins in pathogenicity [3, 18]. However, in this latter virus, non‐canonical P gene mRNA editing was recently found to allow the production of a V‐like protein, whose functional role is currently under investigation [19]. V and W inhibit STAT1 phosphorylation [20], and W binds and sequesters STAT1 and STAT2 proteins in the nucleus through its NTD, thereby inhibiting the interferon (IFN) response [2, 16, 20, 21]. In the absence of IFN stimulation, STAT1/2 shuttles between the cytoplasm and the nucleus. Expression of W causes redistribution of inactive STAT1/2 in the nucleus and prevents STAT1/2 from shuttling back to the cytoplasm. As a result, upon IFN stimulation, the main signal transducers of IFN signaling are absent from the nucleus, therefore preventing the activation of IFN‐stimulated genes.

Additionally, the CTD of the W proteins binds 14‐3‐3 proteins through its serine 449, leading to 14‐3‐3 protein nuclear accumulation. This accumulation enhances the nuclear export of p65, reducing its ability to interact with its promoter targets, thereby inhibiting the NF‐κB‐induced proinflammatory response [22]. Binding to 14‐3‐3 also provokes cell apoptosis through a less defined mechanism [23]. Presently, it is not known whether and how W proteins switch from inhibition of the IFN response to inhibition of the NF‐κB‐mediated response.

We previously showed that the W proteins from HeV and NiV (W^HeV^ and W^NiV^) are intrinsically disordered and can assemble to form highly flexible and curved fibrils [24]. W^NiV^ and W^HeV^ contain cysteine residues implicated in W dimerization [24]. We hypothesized that cysteine residues could impact the fibrillation ability of W proteins. Using various biophysical approaches and site‐directed mutagenesis, we demonstrate here that the cysteine oxidation state dictates the ability of W^HeV^ and W^NiV^ to fibrillate and that nucleation relies on the formation of disulfide bridge‐mediated dimers. We provide the first experimental evidence that W^HeV^ self‐assembles also in cellula, forming various types of condensates in the nuclei of transfected cells. These condensates differ in size and morphology and range from irregular, spherical, and elongated assemblies to giant filamentous structures. In addition, they exhibit various dependencies on the redox state of cysteine residues. Our results also indicate that non‐assembled W^HeV^ is more efficient at blocking STAT1‐mediated IFN response, while W^HeV^ redox‐dependent condensates enhance the repression of the NF‐κB‐mediated antiviral response, suggesting a role in viral escape from the innate immune response.

## 2 Results

### 2.1 Cysteines are Responsible for the Formation of Oligomeric Species and of a Compact Conformation

**FIGURE 1:**
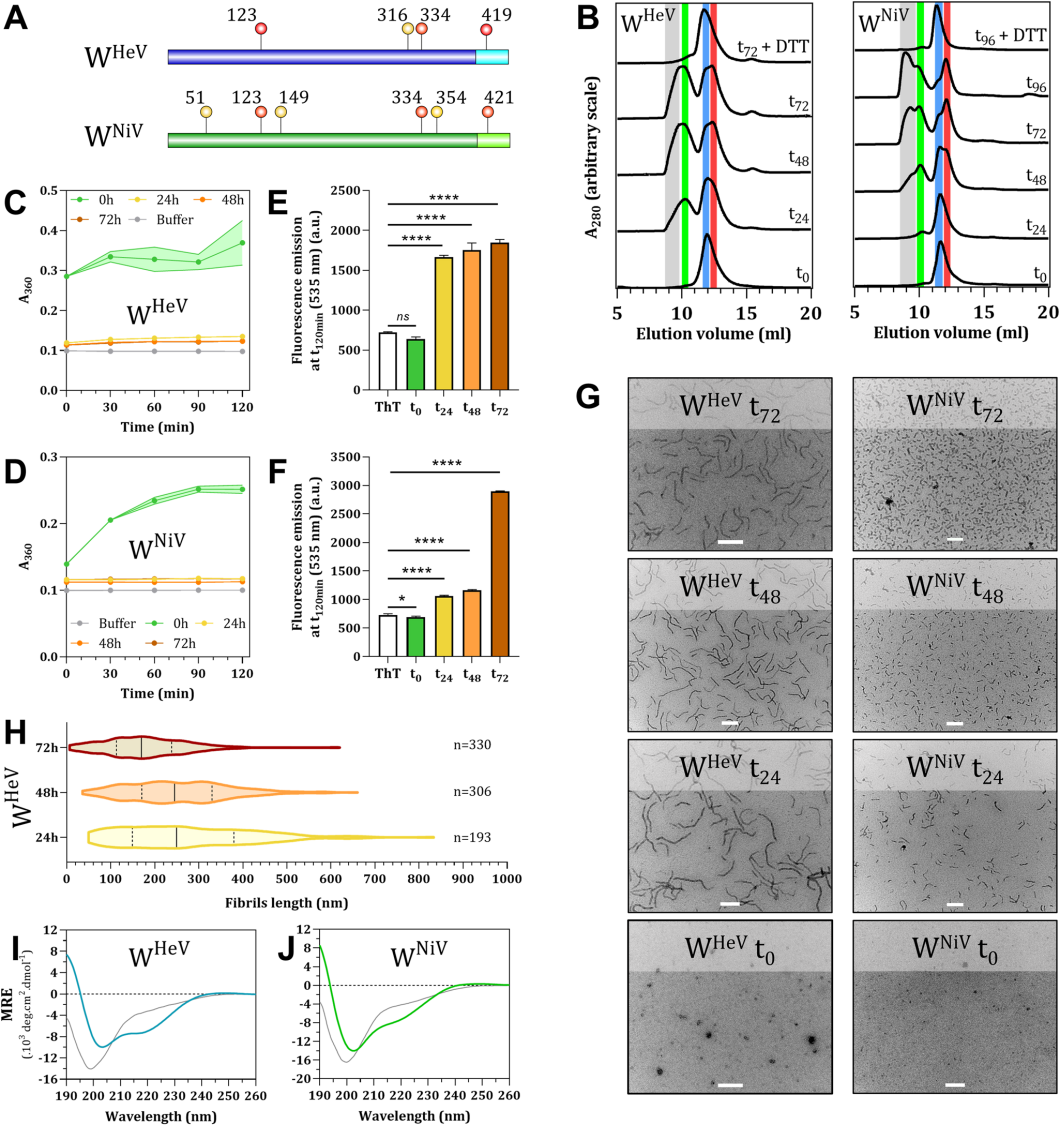
Cysteine residues oxidation of W^HeV^ and W^NiV^ is required to promote their fibrillation. (A) Location of cysteine residues in W^HeV^ (blue) and W^NiV^ (green) proteins. Conserved Cys are indicated in red. Dark colors: N‐terminal domain (NTD), bright colors: C‐terminal domain. (B) Analytical SEC of the W proteins after different preincubation times in oxidative conditions at 37°C in the presence of urea, and either in the absence (t_0_, t_24_, t_48_, t_72_, and t_96_) or in the presence of 2 mm DTT (t_72_ or t_96_ + DTT). The vertical gray, green, and blue bars correspond to the oligomeric, dimeric, and monomeric species, respectively. The vertical red bar corresponds to a redox‐dependent, more compact monomeric form. (C) Turbidity and (E) ThT fluorescence measurements of W^HeV^ preincubated for 0 (green), 24 (yellow), 48 (orange), and 72 h (brown) at 37°C in urea and then buffer exchanged to remove urea. Shown is also the fluorescence of ThT alone (white). Panels C and D show the average values and standard deviation (s.d.) as obtained from *n* = 4 independent measurements. Statistical analysis was made with a one‐way ANOVA, Dunnett's test, ns: not significant, ^****^: *p* <0.0001. The shaded region in (C) corresponds to the error bar. (D) Turbidity and (F) ThT fluorescence measurements of W^NiV^ preincubated or not. The legend is the same as for (C, D). (G) NS‐EM micrographs of W^HeV^ and W^NiV^ incubated for 120 min at 37°C in urea‐free buffer without preincubation (0 h) or following a preincubation of 24, 48, and 72 h at 37°C in urea. (H) W^HeV^ fibril contour length measurements from NS‐EM micrographs after 24, 48, and 72 h of preincubation. Median and quartiles are represented as continuous and dotted lines, respectively. The number of fibril length measurements is indicated for each condition. Scale bar: 200 nm. (I, J) Far‐UV circular dichroism (CD) spectra of isolated W^HeV^ (I) and W^NiV^ (J) fibrils. Data corresponding to the monomeric species are in gray and originate from [25].

The W^HeV^ and W^NiV^ proteins, which we previously showed to be able to form highly flexible and curved fibrils in vitro [24], contain four and six cysteine residues, respectively, three of which are shared by both proteins (Figure 1A). Since the W proteins are intrinsically disordered [24] and thus possess a large solvent‐accessible surface area, we hypothesized a strong reactivity of these residues. The proteins, purified in reducing and denaturing conditions, were incubated at 37°C in the presence of urea to avoid their spontaneous aggregation into fibrils while enabling cysteines to undergo natural oxidation, presumably via the formation of intra‐ and/or interchain disulfide bridges. This pre‐incubation of the proteins in urea will thereafter be referred to as “oxidative preincubation” (Figure S1).

In addition to the monomer (vertical blue line in Figure 1B and peak I in Figure S2A), size exclusion chromatography (SEC) analysis of the W proteins during oxidative preincubation showed the fast formation of dimeric species (vertical green line in Figure 1B), consistent with previous observations [24], as well as the formation of higher‐order oligomers within 24 h for W^HeV^ and 48 h for W^NiV^ (vertical gray line in Figure 1B). The nature of the oligomeric species of W^HeV^ was disentangled by mass photometry, which showed the co‐existence in the sample of monomeric, dimeric, trimeric, and likely tetrameric species (Figure S2B). Additionally, a species (vertical red line in Figure 1B and peak II in Figure S2A) that eluted at a volume slightly larger than that of the initial monomeric species can be detected upon incubation. For W^HeV^, non‐reducing SDS‐PAGE and mass spectrometry analysis revealed that this species corresponds to a monomeric form of the protein (Figure S2C,D), and its elution volume indicates a more compact conformation (Figure 1B).

While W^HeV^ oligomerization does not seem to evolve further after 48 h of oxidative preincubation, in the case of W^NiV^ it continues to progress even after 4 days (Figure 1B). Addition of dithiothreitol (DTT) during the oxidative preincubation in urea (for 72 h for W^HeV^ and for 96 h for W^NiV^), led to the disappearance of both the oligomers (gray line in Figure 1B) and the compact conformation (red line in Figure 1B) indicating that they both are redox‐sensitive and result from the formation of inter‐ and intramolecular disulfide bonds, respectively (Figure 1B).

### 2.2 Cysteine Oxidation is Required for W^HeV^ and W^NiV^ Fibrillation

The aggregation of W^HeV^ and W^NiV^ was followed by turbidity as well as by thioflavin T (ThT) fluorescence measurements (Figure 1C–F). In these experiments, as well as in all the ensuing experiments unless otherwise stated, the W proteins were pre‐incubated for different periods in urea to promote the formation of oligomers as in Figure 1B. After this oxidative preincubation period, urea was removed, and the aggregation process was monitored for 2 h at 37°C in a urea‐free buffer (see procedure in Figure S1). Urea removal without an oxidative preincubation, i.e., at time 0, led to the formation of large and white macroscopically visible insoluble aggregates that resemble precipitated proteins (Figure 1C,D). Aggregates eventually sedimented, and they did not bind ThT (Figure 1E,F). By contrast, oxidative preincubation of W proteins for at least 24 h led to a clear solution (Figure 1C,D) containing species that bind ThT (Figure 1E,F), suggesting the formation of fibrillar structures. After one or two additional preincubation days (48 and 72 h in Figure 1C,E), both the turbidity and the fluorescence emission only poorly evolved for W^HeV^, coincidentally with the arrest of oligomerization as observed by analytical SEC (Figure 1B). In contrast, W^NiV^ ThT binding continued to gradually increase (Figure 1F), mirroring the evolution of its oligomerization (Figure 1B). The nature of the aggregates formed with or without an oxidative preincubation period was assessed by negative‐staining electron microscopy (NS‐EM). Grids were prepared after the 2 h of incubation over which the aggregation and ThT‐binding measurements were carried out (Figure 1G). The insoluble aggregates that are formed without an oxidative preincubation have an amorphous nature, while W^HeV^ and W^NiV^ preincubated in oxidative conditions give rise to fibrils (Figure 1G). Comparison between the far‐UV circular dichroism (CD) spectra of monomeric W^HeV^ and W^NiV^ and the spectra obtained from W^HeV^ to W^NiV^ fibrils (Figure 1I,J) showed a characteristic disorder‐to‐order transition similar to the one observed for the isolated PNT1 region of W^HeV^ (aa 2 to 110) over the course of fibrillation (see Figure 1G in [25]). In particular, the spectra of the fibrils feature a more pronounced negative intensity at 215 nm, indicative of an increase in β‐strand content, although the spectra still exhibit signatures typical of a high intrinsic disorder content (i.e., a pronounced negative peak at 203 nm). This is consistent with the presence of a fuzzy coat around a fibril core enriched in β structure. Based on our previous Fourier‐transform infrared (FTIR) spectroscopy studies carried out on PNT1 fibrils [25], the fibril core presumably encompasses the cryptic amyloidogenic region (CAR, aa 10 to 19) of PNT1.

The constant increase of W^NiV^ oligomers content over time can be ascribed to its higher content in cysteine residues that likely keep reticulating the protein. The balance between fibril nucleation and elongation is low in the case of W^HeV^, as judged from the fact that it rapidly forms fewer, though longer fibrils than W^NiV^. By contrast, W^NiV^ exhibits a much higher nucleation, as judged from the significantly higher number of significantly shorter fibrils detectable on the micrographs taken at various times during the oxidative preincubation, a phenomenon particularly visible after 48 h and, most of all, 72 h of oxidative preincubation (Figure 1G). The ThT fluorescence intensity seems to correlate with the nucleation extent rather than with fibril size (cf. panels E and F with panel G of Figure 1).

W^NiV^ and W^HeV^ fibrils have the particularity of not being straight and rather highly curved (Figure 1G). Measurements of the contour length of W^HeV^ fibrils observed in micrographs revealed that 24 h of oxidative preincubation led to the longest fibrils, up to 830 nm in these experimental conditions, with an average size of 273 nm (Figure 1H). The proportion of short fibrils increased with the incubation time, presumably reflecting fibril fragmentation that regularly occurs in amyloid fibrils. After 72 h of incubation, the average length of fibrils dropped to 179 nm (Figure 1H and Table S1).

Taken together, these observations suggest that disulfide bond formation is the initial step in the fibrillation of both W^HeV^ and W^NiV^ proteins, since non‐oxidized monomeric species are poorly able to undergo fibrillation. To ascertain the hypothesis that oligomers promote the formation of fibrillar structures, we focused on W^HeV^ in light of its lower cysteine content and its faster oligomerization compared to W^NiV^. Since 48 h of oxidative preincubation of W^HeV^ represents a good compromise between solubility and fibril length, and because the oligomerization process does not progress further after this period, this preincubation time was selected for the following analyses of W^HeV^ fibrillation.

**FIGURE 2:**
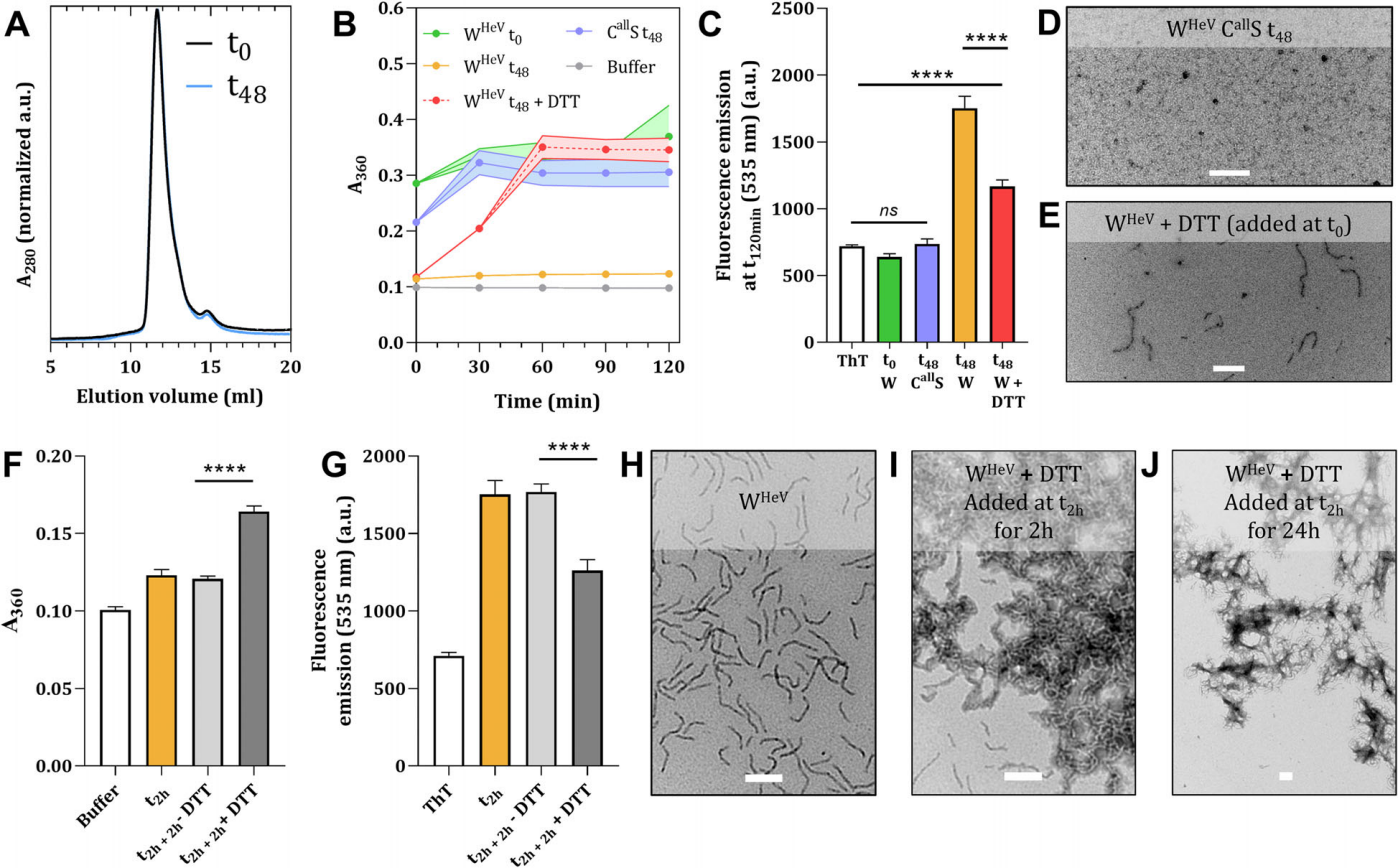
Cys‐to‐Ser substitution abrogates W^HeV^ fibrillation and aggregation in fibrillar structures cannot be reversed by reduction. (A) Analytical gel filtration of W^HeV^ C^all^S preincubated for 48 h (t_48h_, blue) or not (t_0h_, black) in urea. (B) Turbidity and (C) ThT fluorescence measurements of W^HeV^ C^all^S (blue) and of the WT W^HeV^ protein in the presence of DTT (red), both preincubated for 48 h. Non‐incubated (0 h, green) and preincubated for 48 h (orange) WT W^HeV^ data are shown for comparison. NS‐EM micrographs were taken after 2 h of incubation of W^HeV^ C^all^S preincubated for 48 h in urea (D) and of WT W^HeV^ in the presence of 2 mm DTT added after urea removal (E). (F) Turbidity and (G) ThT fluorescence measurements of W^HeV^ preincubated for 48 h and after 2 h of incubation following urea removal (orange), and after 2 extra hours in the presence (dark gray) or absence (light gray) of 2 mm DTT. NS‐EM micrographs of W^HeV^ preincubated for 48 h in urea and after 2 h of incubation following urea removal *plus* 2 extra hours in the absence (H) or presence (I) of 2 mm DTT. W^HeV^ was preincubated for 48 h in urea and then for 24 h in urea‐free buffer in the presence of 2 mm DTT. The solution was centrifuged and the resuspended pellet (J) was observed by NS‐EM. Panels C and G also display the fluorescence of ThT alone (white). Panels B, C, F, and G show the average values and error as obtained from *n* = 4 independent measurements. Statistical analysis was made with a Student's *t*‐test, ns: not significant, ^****^: *p* <0.0001. The shaded region in B) corresponds to the error bar. Scale bar: 200 nm.

The involvement of cysteine residues in W^HeV^ fibrillation was confirmed by generating a variant where the four cysteine residues were all substituted with serine residues (W^HeV^ C^all^S) (Figure 2A–D). As expected, W^HeV^ C^all^S remains monomeric after 48 h of oxidative preincubation in urea (Figure 2A). After urea removal, insoluble aggregates that do not bind ThT were detected, as in the case of the wild‐type (WT) protein not subjected to the oxidative preincubation (Figure 2B,C). Under these conditions, NS‐EM confirmed the sole presence of amorphous aggregates (Figure 2D). Furthermore, the addition of DTT on preincubated W^HeV^ immediately after urea removal, i.e., at t_0_, led to a progressive decrease in solubility over the 120 min of incubation (Figure 2B). After 120 min, ThT binding was weaker in the presence of DTT (red bar) than in its absence (orange bar), but not as low as that observed with the non‐incubated WT protein (green bar) or with the W^HeV^ C^all^S variant (blue bar) (Figure 2C). NS‐EM micrographs taken after 120 min of incubation in the presence of DTT showed the co‐existence of rare and small fibrils, and of amorphous aggregates (Figure 2E).

To further confirm the relation between oligomerization and fibrillation, we artificially generated oligomers by cross‐linking W^HeV^ C^all^S using DTSSP, a cross‐linking agent that contains one NHS ester group at both ends that reacts primarily with primary amines (lysine side chains or N‐termini) (Figure S3). This crosslinker contains a disulfide bond that thus drives the formation of disulfide‐bridged oligomers mimicking those formed by the WT protein. Upon crosslinking, W^HeV^ C^all^S was found to be remarkably soluble during the aggregation measurements (Figure S3A), to strongly bind ThT (Figure S3B), and to fibrillate after 2 h of incubation (Figure S3C). These results, although not conclusive per se, complement and strengthen the ensemble of results described above.

### 2.3 Formation of W^HeV^ Fibrillar Structures is Not Reversible Upon Reduction

To assess whether the fibrillation process was reversible, W^HeV^ was incubated 48 h in oxidative conditions, thus enabling disulfide bond formation, and then allowed to assemble into amyloid‐like fibrils for 2 h after urea removal, before the addition of DTT (Figure 2F–J). Aggregation measurements and NS‐EM were performed after 2 extra hours (t_2h + 2 h_) following DTT addition. As expected, these experiments showed decreased solubility and ThT binding (Figure 2F,G) and the presence of massive clusters of fibrillar structures that were not observed in the absence of DTT (Figure 2, compare H and I).

After 24 h of incubation in the presence of DTT, the samples were centrifuged, and the pellet was resuspended and deposited on an NS‐EM grid (Figure 2J). NS‐EM showed large filamentous structures of micrometric scale consisting of clustered fibrils (Figure 2J), indicating that the fibrillation process is not fully reversible through reduction, though a limited fraction of W^HeV^, presumably monomers detached from the fibrils, became insoluble and amorphous according to the aggregation measurements made just after 2 h of incubation. Thus, while disulfide‐bridged oligomers and/or the redox‐sensitive compact conformation, i.e., species II in Figure S2A, play a critical role in fibril nucleation, once fibrils have formed, a redox‐independent mechanism maintains monomers together. Disulfide bonds also regulate fibril assembly, while their reduction in mature fibrils causes random association of W^HeV^, leading to the formation of fibrillar clusters.

### 2.4 Dimeric Species are Sufficient to Initiate Fibrillation

**FIGURE 3:**
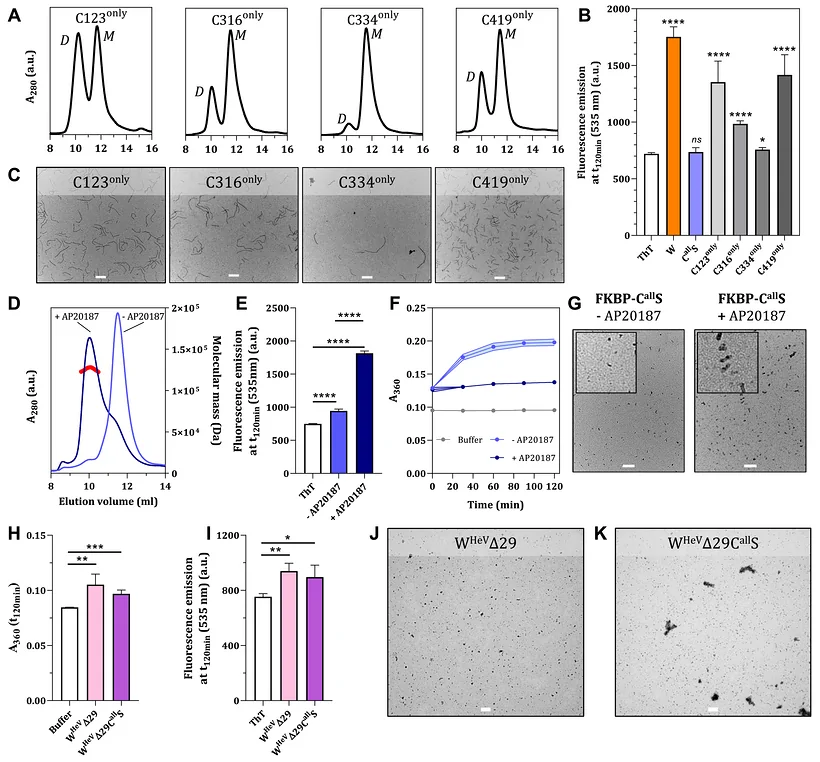
W^HeV^ dimerization is sufficient to promote fibrillation, and the 2–29 region is strictly required for fibrillation. (A) Analytical SEC of W^HeV^ with triple Cys to Ser substitutions preincubated 48 h in urea. M and D stand for monomeric and dimeric species, respectively. (B) ThT fluorescence measurements of W^HeV^ triple variants, preincubated in urea for 48 h, after 2 h in urea‐free buffer. Shown are mean values and s.d. as obtained from *n* ≥ 4 independent measurements. Panel B also shows the fluorescence of ThT alone (white) and of W^HeV^ (orange) and W^HeV^ C^all^S (blue) for comparison. Statistical analyses were performed using the Student's t‐test, comparing fluorescence data to those obtained with ThT alone. (C) NS‐EM micrographs of W^HeV^ with triple Cys to Ser after 2 h of incubation following urea removal. Scale bar: 200 nm. (D) Analytical SEC of FKBP‐C^all^S in the presence (dark blue) or absence (light blue) of the AP20187 molecule along with the molecular mass determination by SEC‐MALLS of FKBP‐W^HeV^ C^all^S in the presence of AP20187 (127 800 Da, red). ThT fluorescence measurements (E) and turbidity (F) of FKBP‐W^HeV^ C^all^S preincubated (dark blue) or not (light blue) with AP20187. Panel E also shows the fluorescence of ThT alone (white). (G) Micrographs of FKBP‐C^all^S after 2 h of incubation with or without AP20187. Inset: zoom. Panels B, E, and F show mean values and s.d. as obtained from *n* = 4 independent measurements. The shaded region in F) corresponds to the error bar. Statistical analysis was made with a Student's *t*‐test; ns: not significant, ^****^: *p* <0.0001. Scale bar: 200 nm. (H) Turbidity and (I) ThT fluorescence measurements of W^HeV^Δ29 (pink) and W^HeV^Δ29 C^all^S (violet) preincubated for 48 h at 37°C in urea and then for 2 h in urea‐free buffer. Shown is also the fluorescence of ThT alone (white). Panels H and I show the average values and standard deviation (s.d.) as obtained from *n* = 4 independent measurements. Statistical analysis was made with Student's *t*‐test, ^*^: *p* <0.05, ^**^: *p* <0.01, ^***^: *p* <0.001. NS‐EM micrographs of W^HeV^Δ29 (J) and W^HeV^Δ29 C^all^S (K) incubated for 120 min at 37°C in urea‐free buffer following a preincubation of 48 h at 37°C in urea.

In order to identify the minimal molecular assembly required for fibril nucleation, W^HeV^ triple Cys to Ser variants were generated, and SEC analysis showed that they are only able to dimerize, although in different proportions (Figure 3A). After 48 h of preincubation in oxidative conditions and a 2 h‐incubation after urea removal, the four variants were shown to be able to bind ThT (Figure 3B) and to fibrillate (Figure 3C), indicating that the sole presence of dimeric species is sufficient to promote W^HeV^ fibrillation. At first glance, the nucleation extent, i.e., the number of fibrils observed on micrographs, seems to be at least partly related to the initial amounts of dimers observed in SEC analysis. Indeed, the W^HeV^ C123^only^, C316^only^, and C419^only^ variants, which display considerable amounts of dimers, give rise to micrographs characterized by a high density of relatively short fibrils. Conversely, the W^HeV^ C334^only^ variant, which weakly dimerizes, displays attenuated nucleation as judged from the presence of rare fibrils in micrographs (Figure 3C). These observations strongly support the implication of dimeric species in the nucleation step of W^HeV^ fibrillation.

Finally, we produced dimeric species by generating a protein construct (FKBP‐C^all^S) whose dimerization can be chemically induced (Figure 3D–G). FKBP‐C^all^S was made by fusing W^HeV^ C^all^S and FK506‐binding protein, FKBP. FKBP is a widely used system to study homodimerization processes since the protein strongly dimerizes in the presence of the AP20187 molecule [26]. FKBP‐C^all^S is expected to be unable to fibrillate in the absence of AP20187, while the addition of the latter should promote the nucleation of fibrils via the formation of a large amount of chemically‐induced dimeric species. After 10 min of incubation in the presence of AP20187, analysis of FKBP‐C^all^S by multi‐angle laser light scattering coupled to size exclusion chromatography (SEC‐MALLS) showed that the chimeric protein was effectively predominantly dimeric (Figure 3D). Urea and unbound AP20187 were removed, and the aggregation and binding to ThT were monitored for 2 h (Figure 3E,F). In these experiments, and in line with expectations, FKBP‐C^all^S turned out to be more soluble and to bind ThT to a greater extent in the presence of AP20187 than in its absence (Figure 3E,F). In the absence of AP20187, the protein slightly binds ThT, an observation that can be accounted for by the initial presence of low amounts of dimeric species in the purified sample. Consequently, a very small number of very short fibrils was observed without AP20187, while their number greatly increased in its presence (Figure 3G). Because of the large molar excess of AP20187 used in these experiments, dimerization is strongly promoted thereby favoring fibril nucleation at the expense of elongation, resulting in numerous and very short fibrils (Figure 3G).

Taken together, these observations demonstrate that W^HeV^ dimerization is the driver of the nucleation process and represents a critical step in the formation of fibrils. A similar conclusion can be drawn for W^NiV^ owing to the correlation between the W^NiV^ oligomers proportion and its nucleation extent (see Figure 1).

### 2.5 The N‐Terminal Region of W^HeV^ Spanning Residues 2 to 29 is Strictly Required for Fibrillation

We recently reported that PNT1 is the most fibrillogenic region among three NTD fragments (PNT1 to PNT3) collectively covering residues 2 to 310 [25]. A predicted CAR within PNT1 was found to be the main driver of fibrillation, with the downstream region encompassing residues 20 to 29 contributing to the process [25]. Removal of the 2–29 region in the context of PNT1 fully abrogated fibrillation [25]. Here, we generated a W^HeV^ variant devoid of the first 29 residues (referred to as W^HeV^ Δ29) as well as the W^HeV^ Δ29C^all^S variant that also lacks cysteine residues. Following an oxidative preincubation, that is, a condition enabling W^HeV^ to form fibrils, and after an additional 2 h incubation in the absence of urea, both variants were found to be slightly insoluble (Figure 3H), to poorly bind ThT (Figure 3I), and to be unable to fibrillate (Figure 3J,K), with micrographs showing only a large number of small assemblies.

Collectively, these data indicate that although oxidized cysteines are necessary, they are not sufficient for W^HeV^ fibrillation to take place. Results enable mapping to residues 2–29 the region strictly required for W^HeV^ fibrillation.

### 2.6 W^HeV^ Forms Different Types of Condensates in the Nuclei of Transfected Cells

We next assessed the ability of W^HeV^ to also form fibrils in cellula. To allow the visualization of W^HeV^ in living cells (i.e., non‐fixed and non‐permeabilized cells), we generated constructs driving the eukaryotic expression of W^HeV^ fused to a tetracysteine (*tc*) peptide tag [27, 28] appended either at the N‐ (*tc*‐W) or C‐terminus of the protein (W‐*tc*). Human embryonic kidney cells (HEK293T) were transfected with these constructs or with an empty control plasmid (EV) or with a plasmid driving the expression of the enhanced green fluorescent protein (EGFP). W^HeV^ was detected by confocal microscopy at 24 h post‐transfection using either the membrane‐permeable ReAsH dye, which fluoresces upon binding to the *tc* tag, or by immunofluorescence (IF) staining targeting W^HeV^.

**FIGURE 4:**
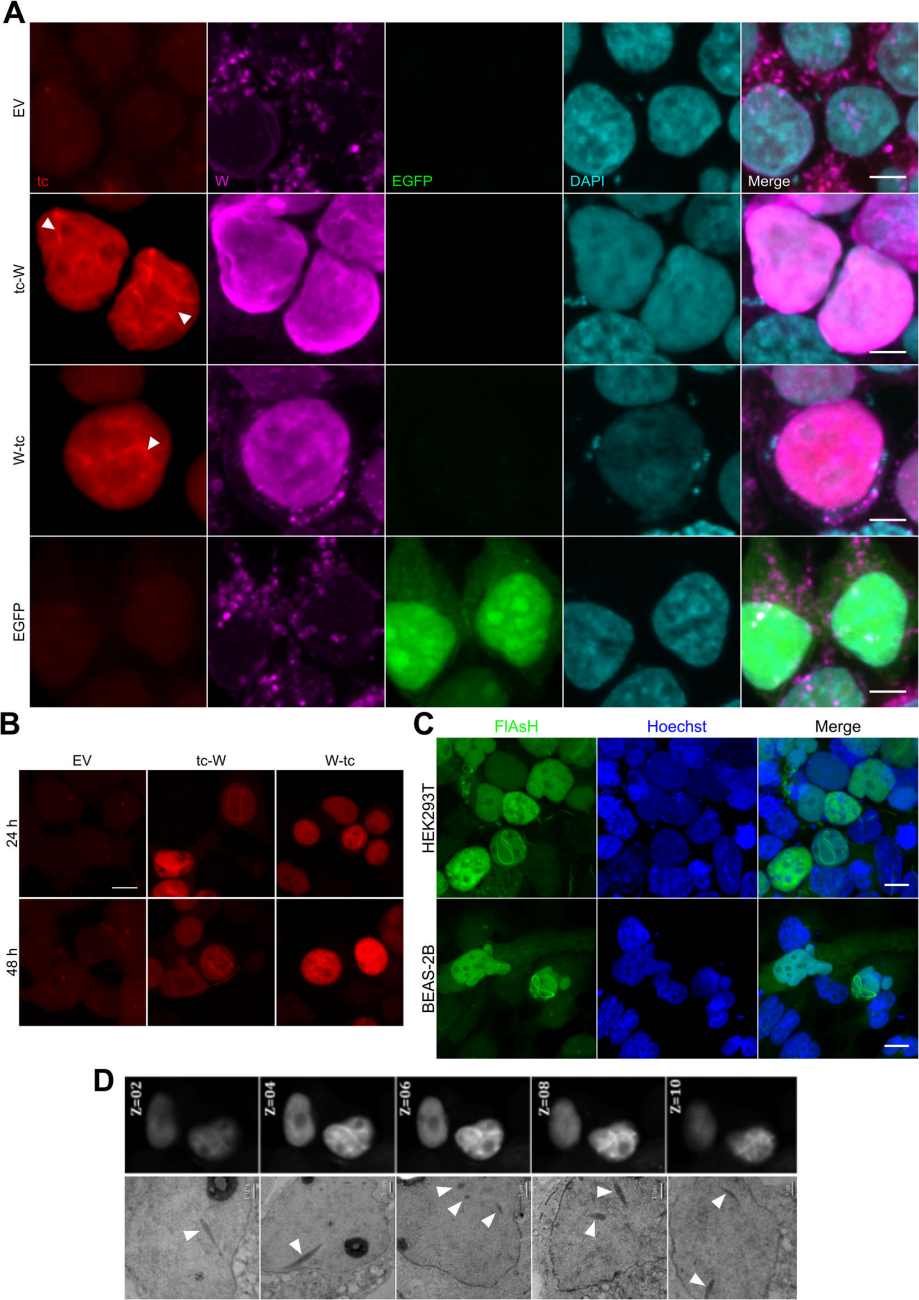
W^HeV^ forms filamentous nuclear structures in cellula. (A) Immunofluorescence and confocal microscopy analysis of HEK293T cells transfected with either an empty vector (EV), enhanced green fluorescent protein (EGFP), or the indicated tetracysteine (tc)‐tagged W^HeV^ constructs. W^HeV^ filaments were visualized 24 h post‐transfection with ReAsH dye to detect the tc peptide (red) and immunofluorescence staining targeting W^HeV^ (magenta). Nuclei were counterstained with DAPI (blue). Scale bar, 10 µm. The white arrow‐heads indicate filaments. (B) Z‐stack projections of live HEK293T cells transfected with an empty vector or the indicated tc‐tagged W^HeV^ constructs. Cells were stained 24 and 48 h post‐transfection with ReAsH dye to detect the *tc* peptide and imaged by confocal microscopy. Images are representative of three independent experiments. Scale bar, 5 µm. (C) Z‐stack projections of HEK293T and BEAS‐2B cells transfected with the *tc*‐W^HeV^ construct. Cells were stained 48 h post‐transfection with FlasH dye and Hoechst. Scale bar, 10 µm. (D) Z‐stack projections of HEK293T cells transfected with the W^HeV^‐tc construct, stained and imaged at 24 h post‐transfection as described in panel B (top). Corresponding electron micrographs of ultrathin sections at equivalent Z positions of the cells shown in the top confocal images. The arrowheads indicate the fibrillar structures detected in the nuclei of the transfected cells (bottom).

In some transfected cells expressing W^HeV^, nuclear filamentous structures along with irregular aggregates could be observed both at 24 and 48 h post‐transfection (Figure 4A,B). Notably, *tc* staining (red) was only visible in W^HeV^‐stained nuclei (magenta), confirming that the filaments are composed of W^HeV^ (Figure 4A). These structures were detectable neither in cells transfected with the empty vector, nor in cells expressing EGFP (Figure 4A), thus ruling out the possibility of non‐specific ReAsH binding. The filamentous structures and irregular aggregates could also be detected upon staining with FlAsH, another *tc*‐specific dye displaying higher contrast and less cytotoxicity in our hands (Figure 4C). Of note, filamentous structures and irregular aggregates were observed in both HEK293T and BEAS‐2B cells, the latter being human bronchial epithelial cells that constitute a relevant cellular model for henipavirus infection that always targets the respiratory tract (Figure 4C).

EM analysis of ultrathin sections of transfected HEK293T cells expressing W‐*tc* at 24 h post‐transfection and displaying ReAsH‐stained nuclear filaments confirmed the presence of nuclear filamentous structures (Figure 4D). These structures were consistently observed at several *z*‐stacks of the reference fluorescence stack (Figure 4D, see Material and Methods and Supporting Information for details of the correlative approach). Similar structures were also observed at 48 h post‐transfection (Figure S4A), while they were never observed in non‐transfected cells (Figure S4B), nor in transfected cells in which no filamentous structures could be detected by confocal microscopy (Figure S4C).

Notably, the nuclear filaments appear to be at least 10 times longer than the fibrils observed in vitro, being in the micrometer, rather than in the sub‐micrometer, length scale (cf. Figures 1H and 4). Unexpectedly, *tc*‐W is significantly more prone to form filaments compared to W‐*tc*, indicating that the *tc* tag interferes with the ability of W^HeV^ to form filaments when present at the C‐terminus of the protein (Table S2). The expression level of W^HeV^ variants was verified by IF staining and appeared to be higher for *tc*‐W^HeV^ compared to W^HeV^‐*tc* constructs (Figure S5), a finding that can be regarded as another plausible explanation for the reduced propensity to form filaments of W^HeV^‐*tc* constructs. In light of this, we focused on the *tc*‐W^HeV^ construct for subsequent analysis, and switched from the pcDNA3.1(+) plasmid backbone to a pCAGGS backbone to drive higher expression levels. Unless otherwise stated, experiments described in the following sections were carried out on HEK293T cells.

**FIGURE 5:**
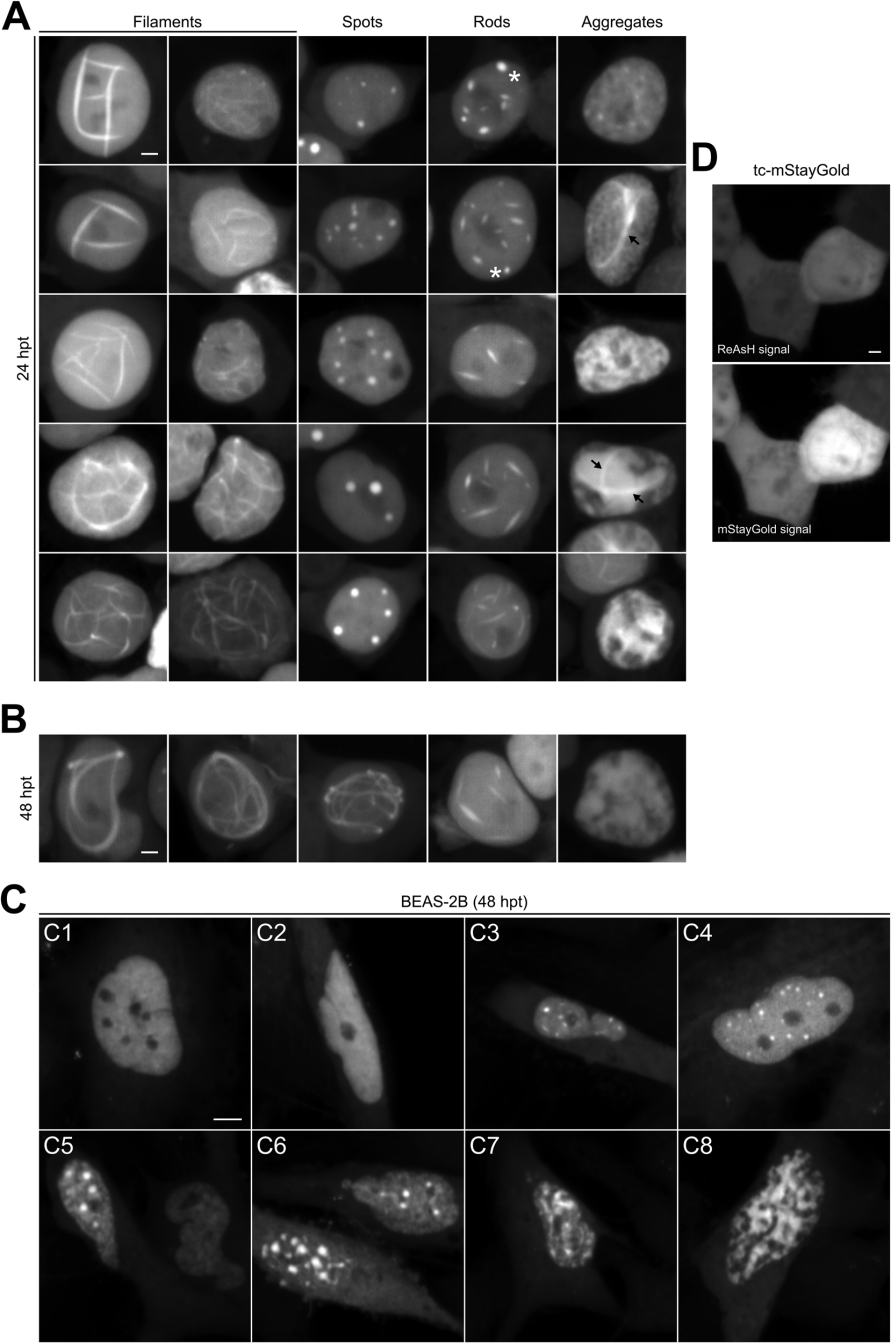
W^HeV^ forms different types of condensates in the nucleus of transfected cells. (A, B) Confocal microscopy imaging of HEK293T cells transfected with *tc*‐W^HeV^ construct and stained with ReAsH at 24 h (A) and 48 h post‐transfection (B). Filaments, spots (globular condensates), rods (short straight structures), and irregular aggregates (sometimes containing filaments, shown by arrows) are detectable in the nuclei. Scale bars, 2.5 µm. (C) Confocal microscopy imaging of BEAS‐2B cells transfected with *tc*‐W^HeV^ and stained with ReAsH at 48 h post‐transfection. Homogenous, diffuse nuclear W (C1, C2), rods (C3), spots (C3, C4, C5, C6), and amorphous aggregates (C7, C8) are detectable in the nuclei. Scale bar, 5 µm. (D) Confocal microscopy imaging of HEK293T cells transfected with a control *tc*‐mStayGold construct and stained with ReAsH at 24 h post‐transfection, showing the absence of condensates and only homogenous, diffuse signal. Scale bar, 2.5 µm.

Beyond filaments with different morphologies, W^HeV^ also forms other types of nuclear condensates (Figure 5). Specifically, at 24 and 48 h post‐transfection, W^HeV^ forms condensates of globular and more elongated shapes (hereafter referred to as *spots* and *rods*, respectively) as well as more irregular aggregates (hereafter referred to as *aggregates*) (Figure 5A,B). The various types of condensates and the filaments are not mutually exclusive. Indeed, they are detectable concomitantly in some cells, as illustrated by the co‐existence of filaments with irregular aggregates (see black arrows in Figure 5A), or spots with rods (see white asterisk in Figure 5A). A similar pattern was observed in BEAS‐2B cells (Figure 5C) and in human lung carcinoma A549 cells (Figure S6). Note that no condensates were observed in cells transfected with a control *tc*‐mStayGold construct (Figure 5D).

**FIGURE 6:**
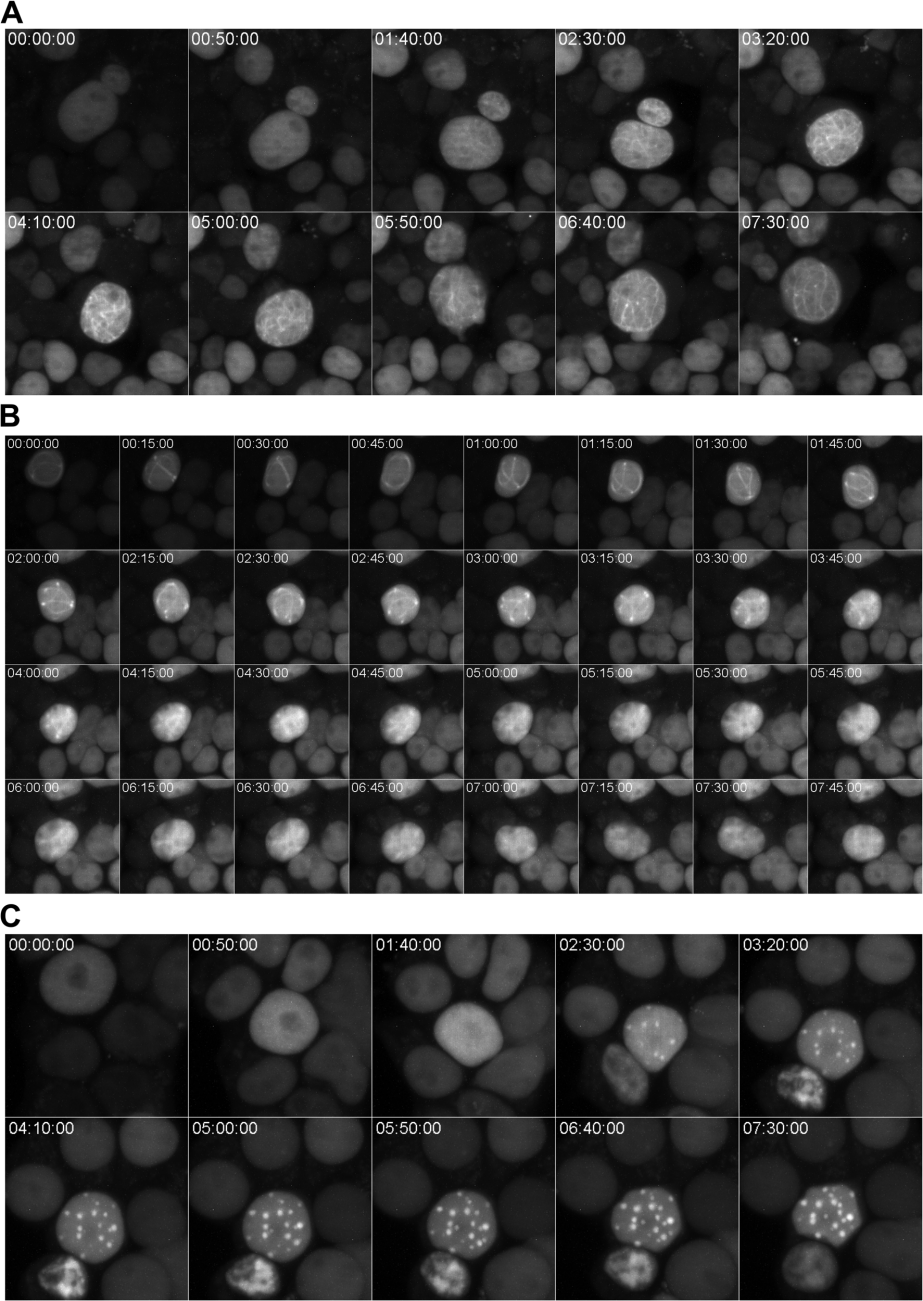
Time‐lapse imaging of transfected HEK293T cells showing the formation and dynamics of W filaments and of the other types of condensates. (A–C) Z‐stack projections of live HEK293T cells transfected with *tc*‐W^HeV^ construct, and imaged by confocal microscopy in the presence of FlAsH, at the indicated times (in the format h:min:sec). (A) Appearance of filamentous structures in the nucleus from an initially homogeneous W signal. (B) Evolution of the morphology of a nuclear filament, which seems to turn into an amorphous structure. (C) Appearance of spots in the nucleus from an initially homogeneous W signal.

In an attempt at unveiling the kinetics of condensate formation, i.e., the temporal relationships among the different types of condensates, we performed time‐lapse confocal microscopy analyses of transfected HEK293T cells and used the milder FlAsH reagent to preserve cellular integrity (Figure 6 and Figure S7). Results support a stochastic scenario for condensate formation: filaments can form from an initially homogeneous and diffuse W signal (Figure 6A), and can then turn into irregular aggregates or seemingly disappear (Figure 6B and Figure S7A), and spots can form from an initially homogeneous W signal (Figure 6C). In other words, and rather unexpectedly, rods and filaments do not correspond to different stages of a “*maturation*” process from spots, and irregular aggregates do not appear to be the last stage of the condensation process. Interestingly, some nuclei display a homogeneous W signal that intermittently transitions into irregular aggregates before reverting to a uniform distribution (Figure S7B, white arrows), indicating that the amorphous aggregation of W is reversible. Overall, our data also highlight that the condensates and especially the filaments are highly dynamic, in that they adopt various conformations throughout the imaging time (Figure 6 and Figure S7A,C).

**FIGURE 7:**
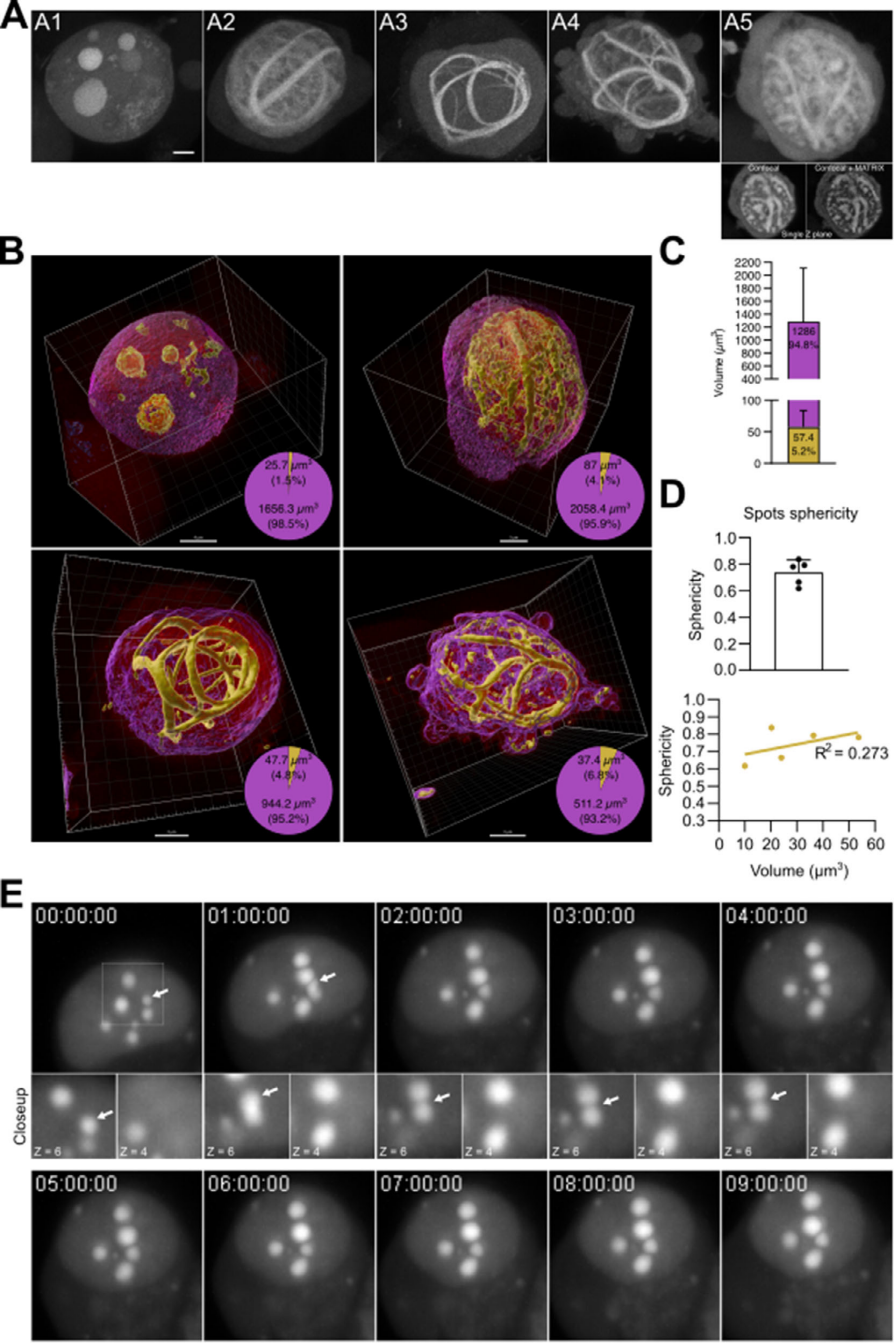
Tridimensional microscopy analysis of W filaments and condensates in transfected HEK293T cells. (A) Z‐stack projections of live HEK293T cells transfected with *tc*‐W^HeV^ construct, stained with ReAsH and imaged by high‐resolution confocal microscopy at 72 h post‐transfection. Scale bar, 2.5 µm. (B, C) Segmentation, visualization, and volume quantification of W structures with Imaris. The pie charts show the volume of the condensates (yellow) and the volume of the nuclei minus the volume of the condensates (violet). (C) Histogram indicating the mean volume of the filaments (yellow) and nuclei shown in panel B. (D) Analysis of the sphericity of the spots shown in panel B. (E) Time‐lapse imaging of transfected HEK293T cells showing the dynamics and partial merging followed by segregation (arrows and closeup of single Z planes) of W spots. Indicated times are in the format h:min:sec.

Based on high‐resolution confocal microscopy acquisitions obtained with transfected HEK293T cells over different Z optical sections (Figure 7A and Movies S1 and S2), the tridimensional (3D) reconstruction of both the globular condensates and the filaments was reconstituted and revealed various morphologies (Figure 7B). These high‐resolution acquisitions and the subsequent condensates segmentation using Imaris software distinctly showed globular spots with the presence of amorphous aggregates (Figure 7A1,B), and long and intertwined filaments, with an estimated section diameter of about 1 to 2 µm, accompanied or not by irregular aggregates (Figure 7A2–A5, B). For filament‐bearing nuclei, 3D reconstruction and segmentation indicate that only one filament with ramifications is present per nucleus (Figure 7B). Quantitative analysis indicated that the globular condensates and the filaments occupy as much as ∼5% of the volume of the nucleus (Figure 7B,C).

We analyzed the sphericity of the spots to ascertain their possible liquid‐like nature (Figure 7D). In the field of liquid–liquid phase separation (LLPS), sphericity is commonly used as a quantitative measure to assess whether a biomolecular condensate behaves like a liquid droplet. A sphericity value close to 1 is indicative of a liquid‐like condensate, as liquids tend to minimize surface area due to surface tension, forming near‐perfect spheres, while values <0.9 suggest solid‐like, gel‐like, or irregular morphology (Hyman et al., 2014). Obtained values support a non‐liquid behavior of the spots (Figure 7D). Besides, the fact that sphericity values and spot volume are not correlated indicates that spots do not become more solid as their size increases (Figure 7D). In further support of a non‐liquid nature, time‐lapse imaging of the spots showed that they exhibit poor dynamics, i.e., they tend to occupy the same position over time, and also poorly coalesce (Figure 7E) (with coalescence being another hallmark of liquid‐like behavior). Indeed, we did not detect any complete coalescence event, but only a few examples of partial merging of spots that eventually segregate again, a behavior consistent with a solid‐like nature (Figure 7E, arrows and closeup).

### 2.7 The Various Condensates Formed by W^HeV^ are Not Associated With Cell Death

**FIGURE 8:**
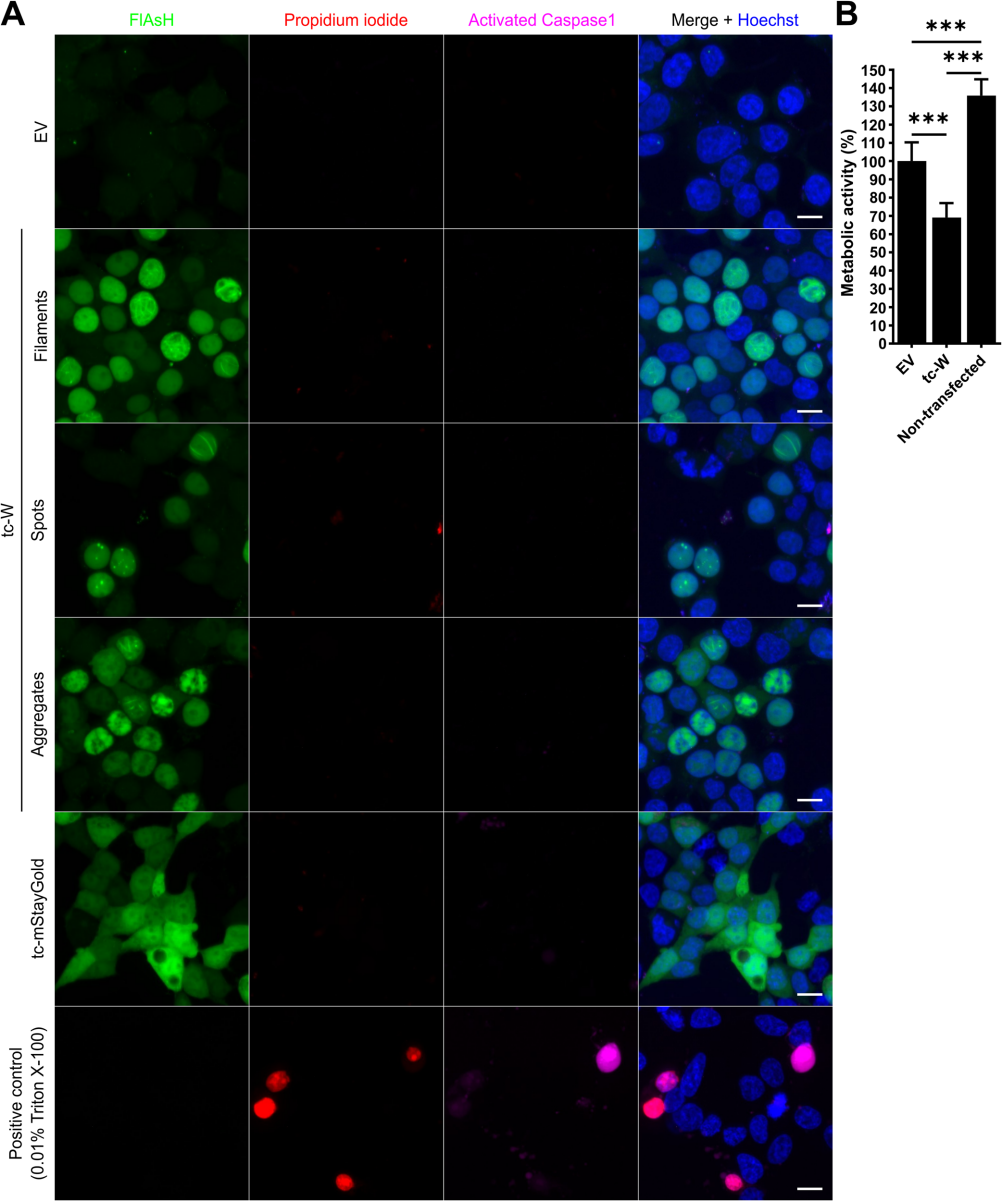
Neither filaments nor the other types of condensates formed by W^HeV^ are associated with cell death features. (A) Z‐stack projections of live HEK293T cells transfected with the indicated constructs, or only treated with 0.01% Triton X‐100, and stained with FlAsH, propidium iodide, Hoechst, and for activated Caspase‐1, and imaged by spinning disk confocal microscopy at 24 h post‐transfection. Scale bar, 10 µm. (B) Analysis of metabolic activity of HEK293T transfected cells with the indicated constructs by fluorometric alamarBlue assay, at 24 h post‐transfection. Data are expressed as mean ± sd and analyzed by one‐way ANOVA followed by a Tukey's multiple comparison test. ^***^ *p* <0.001.

We next investigated the possible impact of the various types of condensates formed by W^HeV^ on cell death and viability (Figure 8). To this end, we stained cells with either propidium iodide or activated Caspase‐1. Propidium iodide is a fluorescent dye that stains DNA in cells with compromised plasma membranes, and is therefore commonly used as a marker for dead or dying cells. Activated caspase‐1 is primarily used as a marker of pyroptosis, a form of programmed cell death associated with inflammation. None of the cells exhibiting W^HeV^ condensates was found to be stained by propidium iodide or caspase‐1 (Figure 8A), indicating that the various condensates induce neither cell death nor pyroptosis. Cells transfected with an irrelevant *tc*‐mStayGold construct or treated with Triton X‐100 served as negative and positive control, respectively. By contrast, alamarBlue metabolic assay, used as a proxy for cellular viability, revealed that transfection with the W^HeV^ expression plasmid was associated with a ∼30% reduction in metabolic activity compared to cells transfected with an empty vector (Figure 8B). Collectively, these data suggest that the observed decrease in cell viability does not reflect cell death, but rather a general metabolic perturbation or reduced cell proliferation following ectopic expression of W.

### 2.8 Removal of the First 29 Residues of W^HeV^ Abrogates Formation of Filaments In Cellula, While Cysteine to Serine Substitution Impairs the Formation of the Other Types of Condensates

**FIGURE 9:**
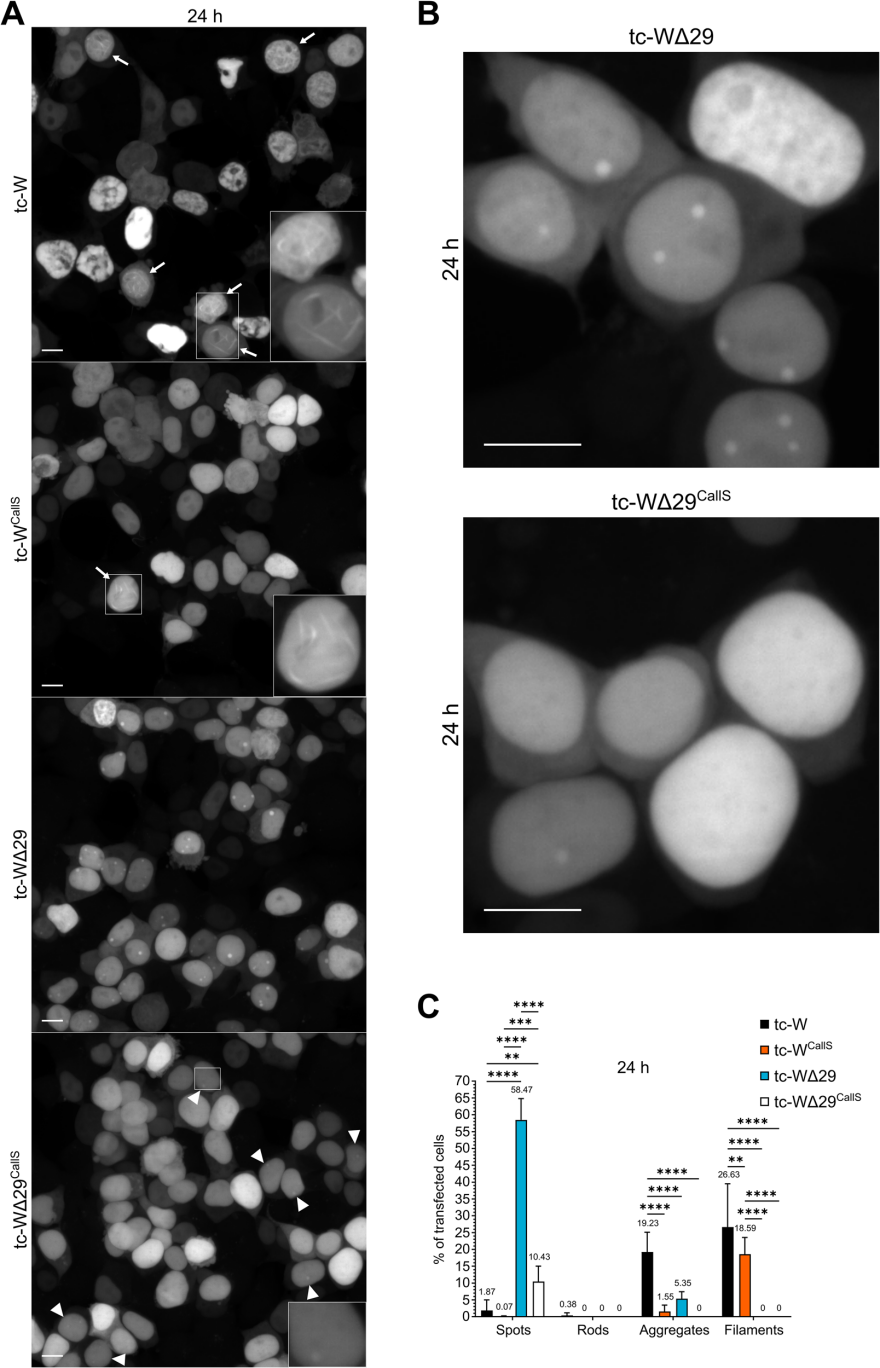
Δ29 deletion strongly impairs W^HeV^ ability to form filaments while CallS substitution impedes formation of non‐filamentous condensates in cellula. (A) Z‐stack projections of live HEK293T cells transfected with the indicated constructs, stained with ReAsH and imaged by confocal microscopy at 24 h post‐transfection. Arrows indicate filament‐containing nuclei, and arrowheads indicate nuclei with small, low‐intensity spots for the WΔ29^CallS^ variant. Scale bars, 10 µm. (B) Closeup of cells expressing the indicated W^HeV^ variants, showing spots and amorphous aggregates for *tc*‐WΔ29, and mostly nuclei with homogenous, diffuse signal for *tc*‐WΔ29^CallS^. Scale bars, 10 µm. (C) Corresponding quantification of the percentage of transfected cells harboring the different nuclear structures formed by W^HeV^ at 24 h post‐transfection. Data are expressed as mean ± sd and analyzed by two‐way ANOVA followed by a Tukey's multiple comparisons test. ^**^ *p* <0.01, ^***^ *p* <0.001, ^****^ *p* <0.0001.

To assess to which extent the phenotype of W^HeV^ variants observed in vitro translates into the cellular context, HEK293T cells were transfected with W^HeV^ constructs driving the expression of N‐terminally *tc*‐tagged W^HeV^ variants in which either all cysteines are replaced with serines (W^CallS^ variant), or the first 29 residues are deleted (WΔ29 variant), or both the deletion and the substitutions are combined (WΔ29^CallS^ variant) (Figure 9A,B). A construct encoding the WT form of the W^HeV^ protein was used for comparison. Quantitative analysis of the condensates formed by the different variants showed that all the variants form very few rods (Figure 9C). Strikingly, WΔ29 does not form any filament at 24 h post‐transfection (Figure 9C). This observation mirrors the inability of this variant to form fibrils in vitro, thus suggesting a potential relationship and/or continuity between fibrils and filaments in spite of the large difference in size. We cannot exclude the possibility that the filaments result from the growth of fibrils and that the latter may escape detection in cellula due to spinning disk confocal microscopy limitations in resolution.

Replacing all the cysteine residues with serines (C^all^S variant) induces a significant decrease in the number of cells with nuclear filaments (Figure 9A,C). However, the most striking feature of this variant lies in its inability to form spots and in its ability to form only very few irregular aggregates (Figure 9A,C). The WΔ29^CallS^ variant is both unable to form filaments and non‐filamentous condensates (Figure 9A–C).

Importantly, no apparent difference in expression level was observed among the various W^HeV^ variants (Figure S8), thus ruling out the possibility that the observed differences could merely reflect differences in expression levels.

Altogether, these data indicate that W^HeV^ can give rise to filaments in cells and that this ability is impaired upon removal of the first 29 residues. The presence of cysteines – presumably oxidized (see below) – is not strictly required for the formation of filaments, as judged from the fact that the C^all^S variant is still able to form filamentous structures. Thus, while in vitro cysteines appear to be necessary, though not sufficient, for fibril formation, they appear dispensable for the formation of nuclear filaments. On the other hand, substituting cysteines with serines abrogates the ability of W^HeV^ to form spots, rods, and, to a lesser extent, irregular aggregates, advocating for a scenario where the formation of these non‐filamentous condensates would be mediated by disulfide bridge‐driven dimers/oligomers.

### 2.9 Both W Expression and HeV Infection Induce Reactive Oxygen Species Production

**FIGURE 10:**
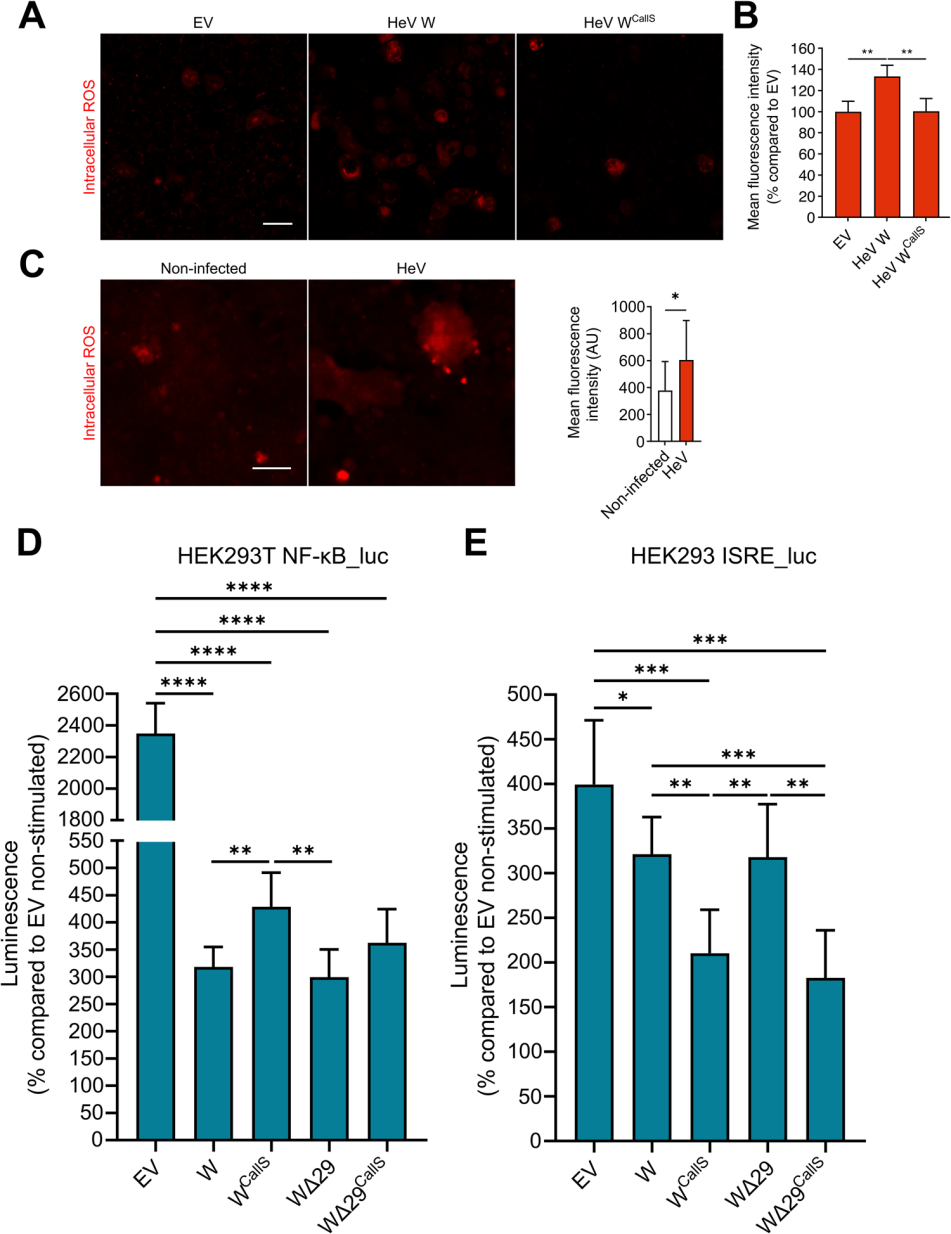
W^HeV^ condensation induces ROS production and impacts innate immunity pathways. (A) Detection of intracellular ROS in W^HeV^‐transfected HEK293T cells at 24 h post‐transfection by confocal microscopy and (B) Fluorescence intensity quantification for each condition. Data are expressed as mean ± sd (*n* = 9 fields of view). Statistical significance was assessed with the Kruskal–Wallis test followed by a Dunn's multiple comparison test. ^**^ *p* < 0.01. Scale bar, 20 µm. (C) Detection of intracellular ROS in HeV‐infected HEK293T cells at 24 h post‐infection by epifluorescence microscopy and corresponding fluorescence intensity quantification for each condition. Data are expressed as mean ± sd (*n* = 9 fields of view). Statistical significance was assessed with the Mann–Whitney test. ^*^ *p* < 0.05. Scale bar, 20 µm. (D) Measurement of NF‐κB pathway activity in HEK293 NF‐κB_luc reporter cells transfected with the indicated constructs. HEK293T NF‐κB_luc cells were transfected and stimulated 24 h later with IL‐1β (50 ng/mL) before assessing NF‐κB activity by luminescence quantification. Data are expressed as mean ± sd. Statistical significance was assessed with unpaired t tests. ^*^ *p* <0.05, ^**^ *p* <0.01, ^***^ *p* <0.001, ^****^ *p* <0.0001. (E) Measurement of IFN response pathway activity in HEK293 ISRE_luc reporter cells transfected with the indicated constructs. HEK293 ISRE_luc cells were transfected and stimulated 24 h later with IFN (100 U/mL) before assessing IFN pathway activity by luminescence quantification. Data are expressed as mean ± sd. Statistical significance was assessed with unpaired *t*‐tests. ^*^ *p* <0.05, ^**^ *p* <0.01, ^***^ *p* <0.001, ^****^ *p* <0.0001.

Oxidative stress is a common feature during viral infections where mitochondria are targeted by viral proteins to promote a strong production of reactive oxygen species (ROS) [29], with this being also true in the case of NiV [30, 31]. As such, henipavirus infection would generate an environment favorable for the formation of disulfide‐bridged dimers/oligomers that mediate the formation of spots, rods and irregular aggregates, as judged from their absence / scarcity in cells transfected with the C^all^S construct (Figure 9). Since these condensates could be detected in the nuclei of cells transfected with *tc*‐W, we expected transfection and/or expression of the W^HeV^ protein to mimic infection and to be able to trigger the production of ROS, thereby enabling ensuing aggregation. Quantification of ROS by confocal microscopy in HEK293T cells transfected with an empty vector or with a construct driving the expression of W^HeV^ confirmed that expression of the latter did significantly induce ROS production (Figure 10A,B). By contrast, transfection with the C^all^S construct did not trigger an increase in ROS (Figure 10A,B). Since the C^all^S variant is still able to form filaments, although to a lesser extent compared to the WT protein, but is severely impaired in its ability to form other types of condensates, results are consistent with a scenario where the “culprits” of the oxidative stress would be the latter rather than the filamentous structures. The increase in ROS triggered by the non‐filamentous condensates would create an oxidative environment further favoring aggregation, thereby amplifying the process. In line with expectations, experimental quantification of ROS upon infection showed an increase in ROS in infected cells (Figure 10C).

### 2.10 W^HeV^‐Mediated Repression of the NF‐κB Pathway is Impaired in the C^all^S Variant that Conversely Supports Enhanced Repression of IFN Signaling

After having shown that W^HeV^ forms various types of condensates in transfected cells, we sought to elucidate their functional impact. W proteins interact with multiple innate immune host factors to counteract the antiviral response (see [16] and references therein cited). We recently showed that W^NiV^ inhibits NF‐κB pathway activation via sequestration of 14‐3‐3 into the nucleus [22]. W proteins also counteract type I IFN (IFN‐I) signaling by sequestering STAT1 into the nucleus [2, 20]. We thus focused on the NF‐κB and IFN‐I pathways in our quest for the condensate functional impact.

To assess the possible impact on the NF‐κB pathway, we used a previously described and validated HEK293T cell line stably expressing a firefly luciferase reporter under the control of a synthetic NF‐κB promoter (HEK293T NF‐κB_luc) [22, 32]. We transfected these cells with the W constructs driving the expression of the various W^HeV^ variants and measured luciferase activity as a proxy for NF‐κB pathway activity. Luciferase expression, strongly induced by IL‐1β treatment, was significantly inhibited in cells transfected with W^HeV^, while inhibition was found to be attenuated for the C^all^S variant (Figure 10D). These results mirror the propensity of these two W constructs to form condensates, with WT W^HeV^ being able to form spots and irregular aggregates and C^all^S being unable to form spots and less able to form irregular aggregates (Figure 9). By contrast, the WΔ29 variant, which is unable to form filaments, behaves like the WT W^HeV^ protein in terms of its ability to inhibit the NF‐κB pathway (Figure 10D), arguing for a lack of impact of filaments on this pathway. The WΔ29^CallS^ variant tends to be less efficient for the repression of the pathway compared to the WT protein and the WΔ29 variant, supporting the critical role of the cysteines.

To assess the possible impact on the IFN response pathway, we used a previously validated HEK293 cell line stably expressing a firefly luciferase reporter under the control of a synthetic Interferon‐Stimulated Response Elements (ISRE) (HEK293 ISRE_luc) [33]. We transfected these cells with the same W constructs described above and measured luciferase activity as a proxy of IFN‐I pathway activity (Figure 10E). Interestingly, the C^all^S variant inhibits the IFN‐I pathway more efficiently than the WT W^HeV^ protein (Figure 10E), displaying therefore an opposite trend with respect to its impact on the NF‐κB pathway (cf. Figure 10D,E).

On the other hand, abrogating the ability to form filaments (WΔ29 variant) has no impact on the W‐mediated inhibition of the IFN‐I pathway, mirroring results obtained when analyzing the impact on the NF‐κB pathway.

Altogether, and as summarized in Table 1, these observations suggest that the W^HeV^‐mediated inhibition of the NF‐κB pathway is associated with the ability of W^HeV^ to form condensates whose formation is mediated by disulfide‐bridged oligomers, and not merely on its presence as a monomeric form. By contrast, the latter condensates would be poorly able to counteract IFN‐I signaling, and the only molecular species able to do so would be the monomeric form of W^HeV^. In other words, while formation of spots and aggregates, but not of filaments, is correlated to an enhanced W‐mediated inhibition of the NF‐κB pathway, favoring a primarily monomeric form of W (via the C^all^S substitution that impedes dimerization/nucleation) enhances the W‐mediated inhibition of the IFN‐I response pathway. Given the unique condensation properties of this variant, it is tempting to hypothesize that its enhanced ability to inhibit the IFN‐I pathway reflects a scenario where the monomeric form of W^HeV^ would be responsible for STAT1 sequestration.

**TABLE 1:** Overview of the in cellula condensation properties and impact on innate immune pathways of the W^HeV^ variants.

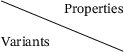	Formation of filaments	Formation of spots	Formation of irregular aggregates	Antagonism of NF‐κB pathway	Antagonism of IFN response pathway
W	++	+	++	++	+
W^CallS^	+	−	+	+	++
WΔ29	−	+++	++	++	+
WΔ29^CallS^	−	++	+/−	+	++

### 2.11 STAT1 Strongly Binds W Monomers but Not Fibrils

**FIGURE 11:**
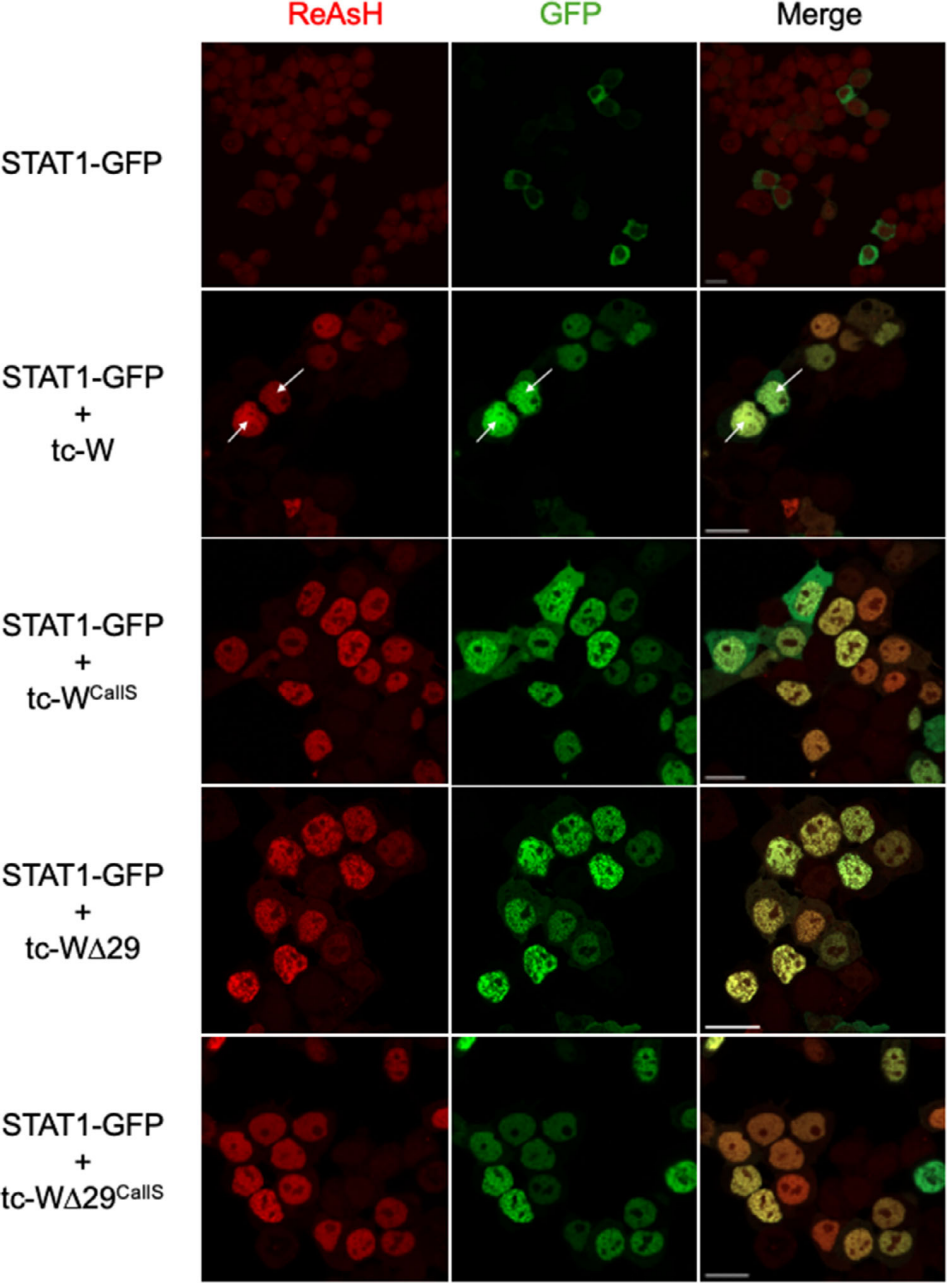
Confocal microscopy images of HEK293T cells transfected with STAT1‐GFP alone (control) or STAT1‐GFP *plus* the indicated tc‐W variants. Cells were stained with ReAsH and fixed with 4% PFA at 24 h post‐transfection. The white arrows indicate filaments. Scale bar: 20 µm.

In order to experimentally investigate the W assembly state dependence of STAT1 binding, we first assessed the ability of the various W condensates to interact with STAT1 in cellula. To this end, we used HEK293T transfected cells expressing various tc‐W variants and a STAT1‐GFP fusion combined with confocal microscopy to investigate the co‐localization between the different W condensates and STAT1 (Figure 11). When expressed alone, STAT1‐GFP displayed a predominantly diffuse cytoplasmic distribution. In contrast, in the presence of the tested W variants, STAT1 showed clear nuclear co‐localization with W condensates in all examined cells, indicating that W and its variants relocalize STAT1 to the nucleus, presumably prior to its phosphorylation since the experiment was performed in non‐stimulated cells.

These data suggest a similar ability of the different W condensates to bind STAT1, in contrast with the hypothesis that the enhanced ability of the C^all^S variant to inhibit the IFN‐I pathway stems from a better ability to bind STAT1. It should be underscored however, that overexpression of both STAT1 and W proteins may hamper the detection of subtle differences since a large amount of W^HeV^ may remain monomeric.

**FIGURE 12:**
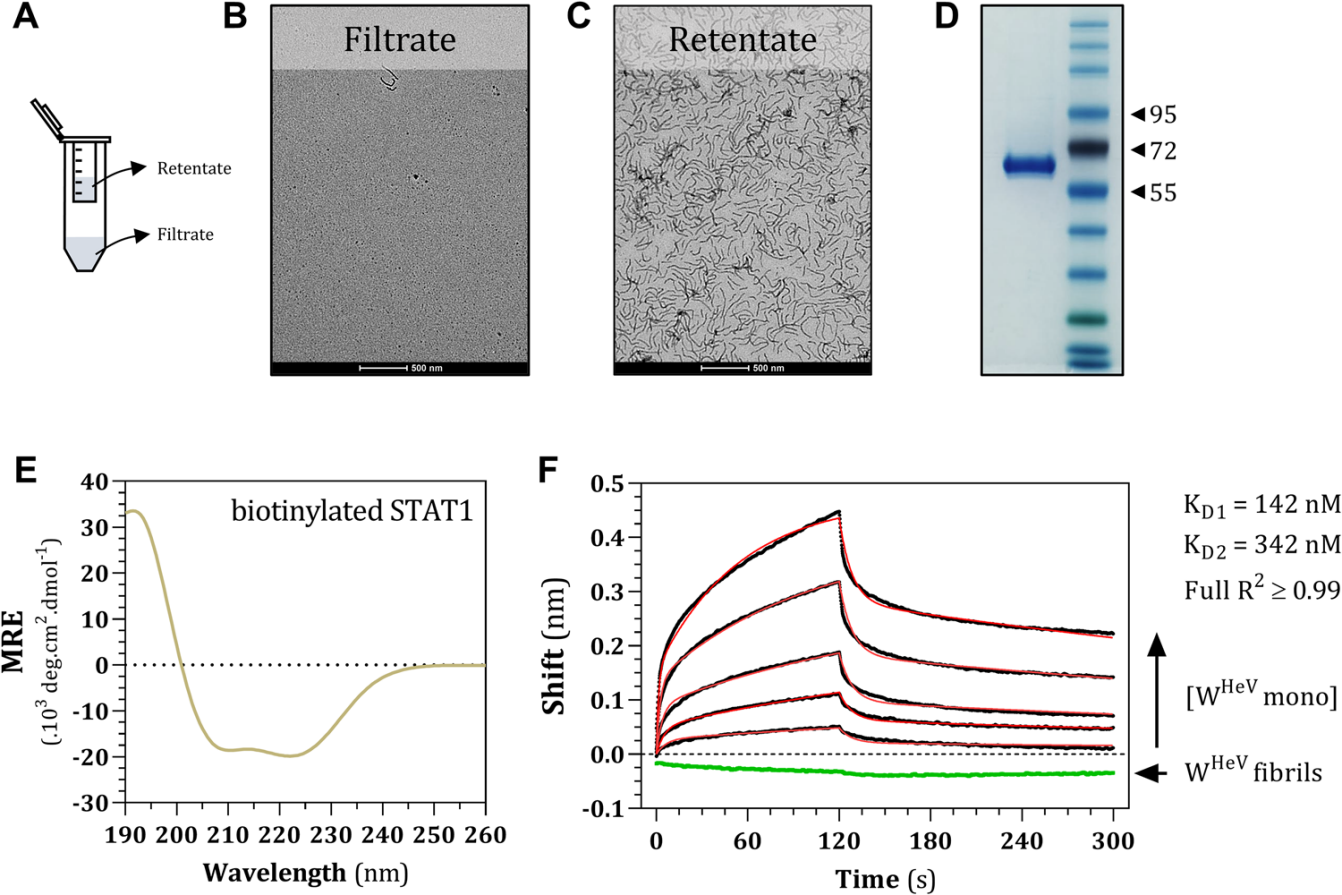
STAT1 interacts with monomeric W^HeV^ but not with its fibrillar form. (A) Schematic representation of a concentrator device used for fibril enrichment and its two fractions (filtrate and retentate). 3.5 µL at a concentration of 5 µm of either the filtrate (B) or the retentate (C) were deposited on grids and observed by NS‐EM. Scale bar: 500 nm. (D) SDS‐PAGE analysis of 4 µg of purified STAT1_132‐682_ in reducing conditions. Proteins were stained with Coomassie blue (molecular markers: NEB, P7719). (E) Far‐UV CD spectrum of biotinylated STAT1_132‐682_. (F) BLI experiments using immobilized STAT1_132‐682_ dipped into various concentrations of either monomeric (mono) species of W^HeV^ (black dots, from 0.1 to 1.6 µm), or of a single concentration of W^HeV^ fibrils (green dots, 1.6 µm). Full red curves: sensorgrams fitting using a heterogeneous ligand model (2:1). Equilibrium dissociation constants (K_D_) and fitting quality (R^2^) are indicated.

We therefore resorted to also performing in vitro interaction studies using biolayer interferometry (BLI). To this end we first enabled W^HeV^ to form fibrils and then isolated them using a concentrator (Figure 12A). The fibril enrichment was assessed by NS‐EM that clearly demonstrated a high quantity of fibrils in the retentate compared to the filtrate fraction (Figure 12B,C). A recombinant fragment of human STAT1 encompassing residues 132–682, (hereafter referred to as STAT1_132‐682_), previously shown to be able to bind to the 92–190 region common to the NiV P, V and W proteins [34], was purified and biotinylated to enable immobilization on streptavidin biosensors (Figure 12D). The foldedness of the biotinylated recombinant STAT1_132‐682_ protein was verified by CD, which showed the signature typical of a folded protein enriched in α‐helices, in accordance with its known 3D structure (pdb code 1YVL) (Figure 12E).

Either the monomeric (unincubated and extemporaneously thawed W^HeV^ protein) or its isolated fibrillar form was used as analytes for BLI experiments. Interestingly, a strong interaction was detected with the monomeric species of W^HeV^ but not with its fibrillar form (Figure 12F). The best sensorgram fitting was obtained using a 2:1 model (W^HeV^:STAT1) (Figure S9), suggesting the presence within W^HeV^ of two STAT1 binding sites with equilibrium dissociation constants (K_D_) of 142 nm (ka_1_ = 1.05 × 10^4^ m ^−1^. s^−1^; kd_1_ = 1.5 × 10^−3^ s^−1^), and 342 nm (ka_2_ = 3.58 × 10^5^ m ^−1^. s^−1^; kd_2_ = 1.22 × 10^−1^ s^−1^) (Figure 12F). The complex only partially dissociated, suggesting that a population of STAT1 remains tightly bound to W^HeV^, in agreement with its role in trapping STAT1 intracellularly (Figure 12F).

Results therefore suggest that STAT1 binds much better (if not exclusively) the monomeric form of W^HeV,^ providing direct experimental support for a scenario where the STAT1‐binding site would be occluded in fibrillar W^HeV^. This observation thus provides a mechanistic framework to rationalize the enhanced ability of the C^all^S variant to inhibit the IFN‐I response pathway.

## 3 Discussion

We herein report the crucial implication of cysteine residues in the fibrillation ability of the W protein in vitro. SEC analysis of W proteins preincubated for different periods under denaturing and oxidative conditions showed that they undergo disulfide bridge‐mediated oligomerization. This process is closely related to W^HeV^ and W^NiV^ solubility since, in the absence of disulfide‐bridged oligomers, amorphous aggregates are formed, as shown by experiments in the presence of a reducing agent or using the W^HeV^ C^all^S variant. Oligomerization of both W^HeV^ and W^NiV^ proteins was also found to be closely tied to the formation of fibrils, and as such, oligomers represent the fundamental drivers of primary nucleation. The requirement for a single cysteine residue for fibril formation and the substantial increase in W^HeV^ C^all^S solubility and ability to form fibrils upon generation of chemically‐induced dimers support the conclusion that dimeric species are the primary drivers of nucleation. Notably, those dimers can be formed through any cysteine residue, as judged from the fibrillation abilities of the Cys123^only^, Cys316^only^, Cys334^only^ and Cys419^only^ variants. Furthermore, dimers do not need to be disulfide‐bridged to promote nucleation, as illustrated by the fibrillation abilities of the artificially induced C^all^S dimers as obtained via addition of AP20187 to FKBP‐C^all^S. Nucleation via disulfide‐bridged dimers has been described in the case of Tau [35] and fish β‐parvalbumin [36], while non‐covalent dimers were shown to initiate nucleation of other fibrillogenic proteins, such as the Aβ peptide [37] or α‐synuclein [38, 39]. However, unlike these latter proteins, the monomeric species of W^HeV^ and W^NiV^ are not stable in solution and rapidly aggregate. W^HeV^ and W^NiV^ disulfide‐bridged oligomers seem to enable bypassing the lag phase that is usually observed during the amyloidogenesis process [40] through the formation of covalent oligomers that eventually polymerize. Interestingly, mature W^HeV^ and W^NiV^ fibrils are not completely sensitive to reduction, indicating that a successive and distinct stabilizing mechanism, presumably brought by the PNT1 region (and in particular by the residues 2–29) [25], is at play in the fibril elongation step compared to the initial nucleation step.

Another consequence of the presence of multiple Cys in W^HeV^ is the formation of intrachain disulfide bonds, which decreases the hydrodynamic radius of the monomer. This compact conformation is not required for W^HeV^ fibrillation, as shown by the Cys^only^ W^HeV^ variants. On the contrary, intrachain disulfide bonds may impair the fibrillation process by decreasing the nucleation extent since fewer cysteine residues are available to establish an interchain bridge. This scenario is in agreement with previous observations on other fibrillogenic proteins, where increasing the compaction of α‐synuclein via the addition of an artificial disulfide bond strongly decreases its fibrillation propensity [41]. Similarly, intrachain disulfide bonds in 4R Tau isoforms cause the formation of compact monomers that are not able to fibrillate [35]. According to our observations, the probable function of cysteine residues is 1) to allow monomers to dimerize, which will enable fibril nucleation possibly by reducing the entropy of the disordered chains, at least when several Cys are oxidized, 2) to maintain them soluble and 3) to prevent fibril clustering.

As shown herein and in previous studies [42], fibrils made of W are extremely curved. Their curved nature raises the question as to whether they possess the typical cross‐β organization of classical amyloids. FTIR studies carried out on the similarly highly curved fibrils made of the PNT1 fragment of W^HeV^ (aa 2–110), showed that they are enriched in cross‐β structure [25]. Since PNT1, and, more specifically, the region encompassing the first 29 residues, is the major driver of fibrillation, and since CD spectra of W^HeV^ show an increase in β structure content with respect to the monomeric form, it is plausible that findings obtained with PNT1 fibrils can be transposable to fibrils made of W, although definite answers await high‐resolution structural data.

The highly curvy nature of fibrils and their heterogeneity suggests that these are functional amyloids, i.e., fibrillar assemblies that differ from the pathological amyloid fibrils typically observed in amyloidoses, and that are rather of functional relevance to the virus [43]. Indeed, functional amyloids can exhibit distinct fibril morphologies — including heterogeneous and curved fibrillar forms — that reflect differences in assembly dynamics and stability compared with many pathological amyloid fibrils [44].

Using a tetracysteine tag already successfully employed in [45], we showed that in cellula W^HeV^ forms different types of condensates. It should be noted that although LLPS is often discussed synonymously with biomolecular condensation, the latter is in fact more general, covering processes beyond just liquid–liquid demixing and including gelation, crystallization, polymerization, clustering, and amorphous or amyloid aggregation [46]. W^HeV^ was found to form giant filamentous structures with exclusive nuclear localization. In light of the differences in size, filaments cannot be regarded as the direct cellular counterpart of fibrils observed in vitro. However, one can speculate that giant filaments are composed of bundles of fibrils, a hypothesis that would need to be ascertained using high‐resolution ultrastructural analysis, as described for the fibrillar condensates formed by the NSs protein from the Rift Valley fever virus (RVFV) [45]. In addition, the WΔ29 variant is unable to form filaments, a finding that parallels the failure of this variant to fibrillate in vitro, thereby supporting the hypothesis of a close relationship between filaments and fibrils.

Fibril formation in vitro appears to take place through the *deposition* pathway rather than through the *condensation and maturation* pathway [47], as judged from the lack of evidence in support of a droplet state preceding the formation of fibrils. Mirroring these findings, filaments observed in cellula do not appear to result from *maturation* of globular condensates. Incidentally, the latter were found to have a seemingly solid‐like nature. Whether globular condensates contain fibrils of the same length as those observed in vitro will be investigated in future studies.

Substitution of cysteine residues with serines (C^all^S variant) impairs formation of non‐filamentous condensates (spots and irregular aggregates), whose formation therefore appears to be mediated by disulfide bridge‐driven dimers/oligomers. The reliance on oxidized cysteines for fibril formation in vitro raises the question of whether W proteins can form condensates mediated by disulfide bridges in cellula, given the reducing environment of the nucleus (and of the cytosol). Notably, NiV infection induces ROS production [31] as does HeV infection (Figure 10C), and we showed here that the sole ectopic expression of W^HeV^ resulted in the production of ROS, thereby creating an environment favorable to the formation of condensates relying on the formation of disulfide bridges and eventually provoking and/or amplifying W^HeV^ condensation. In addition, *Henipavirus* virions contain W and V proteins [11], and one can imagine that during budding the viral particles bring other elements from the infected cell that favor oxidative stress upon infection of naïve cells.

These observations are in line with several studies on various proteins whose condensation in fibrillar structures relies on disulfide bridges. The human protein p16^INK4A^, which only fibrillates when its unique cysteine is oxidized, was shown to fibrillate in cultured cells under mild oxidizing conditions [48]. Likewise, disulfide bond‐dependent viral amyloids made by the RVFV NSs protein could be observed in both transfected and infected cells [45]. More recently, the cryo‐EM structure of ex vivo amyloid fibers made by the human atrial natriuretic peptide (ANP) showed notable similarities with W^HeV^. In particular, ANP exhibits disulfide bond‐dependent fibrillation, with no specific disulfide bond being required for fibrillation and with several different dimeric organizations giving rise to amyloid fibrils equally [49]. These three examples, together with the ones presented herein, indicate that the oxidation of cysteine residues shown to be critical for protein condensation does take place in the cellular context.

During viral infections, the increase in ROS can cause oxidative shifts in glutathione redox potential in the order of ∼20–70 mV compared with basal reducing conditions (≈−300 mV) [50]. Although no quantitative data are available on the nuclear redox potential increase induced by viral infection, in mammalian cells, nuclear redox‐related proteins like TRX1 (thioredoxin‐1) have been observed to undergo ∼60 mV oxidative shifts from a baseline of ∼−300 mV under conditions of excess ROS [51]. This increase in redox potential is sufficient to impact signaling, transcription factor activity, and redox‐sensitive protein domains, and hence presumably also enables W protein cysteine oxidation and ensuing condensation.

Finally, it is worthy to note that cysteine oxidation and subsequent disulfide bridge formation can also be metal‐catalyzed, as already reported for copper [52]. Copper exists in two main oxidation states in cells: Cu(I) (cuprous) and Cu(II) (cupric). This redox cycling allows copper to participate in reactions that transfer electrons. In the presence of oxygen (O_2_) and reducing molecules like cysteine, glutathione (GSH), or other thiols, copper can catalyze oxidation reactions via Fenton‐like chemistry leading to the formation of disulfides. Copper interacts strongly with thiols (e.g., GSH and cysteine) and forms Cu‐thiolate complexes that make thiol oxidation more favorable even at physiological potentials. Concomitant production of H_2_O_2_ further accelerates oxidation processes [53]. The stress‐induced increase in oxidation potential, combined with the presence of copper, may therefore act in concert to enable cysteine oxidation.

In NiV‐infected cells, equimolar proportions of P, V, and W transcripts are detected in the early stage of infection while the quantity of V and W transcripts increases as the infection progresses [13]. V and W proteins are therefore synthesized at an early stage after infection. The quick formation of W condensates may benefit the virus to fight against the cell innate immune response in light of the short timescale of viral infections, the duration of which is limited to a few hours in vitro [3] and a few days in vivo after experimental infection [54].

As previously described [22], W^NiV^ CTD binds and accumulates 14‐3‐3 into the nucleus, leading to an increased cytosolic translocation of NF‐κB p65/p50 and consequently a reduced expression of proinflammatory cytokines. Our study confirms and extends these findings: not only does it show that the W^HeV^ protein is able to impair the NF‐κB pathway, but it also sheds light on the molecular underpinnings. Indeed, the formation of spots and irregular condensates was shown to augment this ability, as judged from the observation that the NF‐κB pathway is less impaired in the C^all^S variant that is unable to form this type of condensate in cellula. By contrast, it is the monomeric form of W^HeV^ that appears to be more efficient at counteracting IFN‐I signaling. As such, the virus would need both non‐assembled and self‐assembled forms of W to ensure efficient blockade of the host's innate immune response. It is noteworthy that differential functional outcomes depending on the condensation states (monomeric, oligomeric, fibrils, etc.) are also reported for other self‐assembling proteins. For instance, pathogenic assembly of α‐synuclein gives rise to different types of condensates in neurons, among which are oligomeric species that are causative of neuronal insults and toxicity. Interestingly, these oligomeric species are released by α‐synuclein fibrils, with short fibrils being consequently more neurotoxic than long ones because of their higher proportion of fibril endings [55]. In vivo, variants of α‐synuclein that form oligomers are neurotoxic, contrary to variants inducing fibril formation that are far less toxic [56]. Likewise, the forms of W endowed with functions appear so far to be the monomeric and the oligomeric species, but not so much the filamentous species.

Contrary to RVFV, whose disulfide‐bridge‐mediated NSs large filaments were shown to be associated with IFN‐I response silencing [45], the functional impact of W^HeV^ filaments remains elusive and deserves future investigation. The herein uncovered impact of W^HeV^ redox‐dependent, non‐filamentous condensates on the NF‐κB pathway aligns with previous reports pointing out the critical role of viral condensates in inhibition of the antiviral response (refer to [57] as an example) while further broadening the biological relevance of biocondensates in host immune evasion to encompass non‐liquid assemblies.

In conclusion, the present study constitutes the first experimental evidence of the ability of the *Henipavirus* W proteins to self‐assemble in a cellular context. By establishing a functional link between redox‐dependent condensates and impairment of the NF‐κB pathway, this study provides the first clues on the functional impact of these latter condensates. In addition, by showing a link between W oligomerization and reduced ability to inhibit the IFN‐I response, our study provides hints on the mechanisms underlying STAT1 sequestration, a key determinant of *Henipavirus* pathogenicity. Our study thus contributes to shedding light on the possible molecular mechanisms by which this virulence factor may contribute to *Henipavirus* pathogenesis. Further studies will be necessary to explore to what extent W condensation participates in W function and potentially in the switch from inhibition of the IFN‐I pathway to inhibition of NF‐κB signaling in the context of *Henipavirus* infection. The present study sets the stage for future investigations aimed at assessing the generality of this mechanism in other paramyxoviruses expressing a P‐encoded W protein. From a more general perspective, it also adds to the hitherto relatively few examples of viral fibrillar and non‐fibrillar functional condensates and thus broadens our knowledge on their use by viruses as a strategy to counteract host cell functions.

## 4 Experimental Section

### 4.1 Generation of Constructs

The generation of the constructs is detailed in the Supporting Information, Experimental section (see Tables S3 and S4). The resulting constructs for bacterial and eukaryotic expression allow the expression of 6‐His tagged recombinant W proteins and of N‐ or C‐terminally *tc*‐tagged proteins, respectively. The bacterial expression pET28 construct encoding residues 132–682 of human STAT1 with a C‐terminal Strep‐Tag II was a kind gift of Marc Jamin (Institut de Biologie Structurale, Grenoble, France).

### 4.2 Expression and Purification of Recombinant Proteins

Expression of all recombinant proteins was performed in the *E. coli* strain T7pRos. The detailed protocol for protein expression and purification of W proteins and of STAT1_132‐682_ is detailed in the Supporting Information, Experimental section. Briefly, recombinant W proteins and their variants were purified in denaturing and reductive conditions (i.e., in the presence of 6 m urea and of 10 mm DTT) by Ni‐NTA affinity chromatography and anion exchange chromatography (AEC). Protein samples were then desalted to remove the DTT and stored at −80°C in denaturing conditions. FKBP‐ C^all^S was purified in the same way except that an additional preparative SEC was performed in 20 mM HEPES, 150 mM NaCl pH 7.2 (HBS) supplemented with 1 m urea. Purified proteins analyzed by SDS‐PAGE are shown in Figure S10.

### 4.3 Protein Sequencing by Tandem Mass Spectrometry

The identity of the compact conformation of W^HeV^ was confirmed by mass spectrometry analysis of tryptic fragments obtained after digestion of the purified protein band excised from SDS‐polyacrylamide gel. The detailed protocol is further described in Supporting Information, Experimental section.

### 4.4 Analytical SEC of Non‐Incubated and Incubated Proteins

One hundred µL of W^HeV^ (WT and variants) or W^NiV^ (WT) samples at 75 µm in HBS/6 m urea buffer, incubated at 37°C without agitation for different times, were injected onto a Superdex S200 Increase 10/300 GL (Cytiva) column equilibrated in HBS and eluted in the same buffer at a flow rate of 0.8 mL/min and at room temperature.

### 4.5 Mass Photometry Measurements

Mass photometry experiments were carried out on a TwoMP mass photometer (Refeyn). WT W^HeV^ was incubated at a final concentration of 75 µm for 48 h at 37°C and then diluted to 100 nm in HBS for molecular mass measurements.

### 4.6 Turbidimetry and Fluorimetry Measurements

Protein samples were incubated at 37°C for different times (indicated in each experiment) as detailed in the Supporting Information, Experimental section. Protein solutions were then buffer exchanged to remove urea. The resulting urea‐free protein samples were analyzed in the presence of thioflavin T (ThT). Turbidity measurements and fluorescence measurements were performed on a Tecan microplate reader GENios Plus in black 96‐well plates with transparent flat bottom (Greiner, 655096) incubated at 37°C.

The same protocol was followed in the experiments made in the presence of DTT. 2 mm DTT was added either just after the buffer exchange step (t_0_), or on a W^HeV^ sample that had already been incubated for 2 h (t_2h_). In this latter case, turbidity and fluorescence were monitored for 2 extra hours (t_2h + 2 h_). This latter sample was further incubated at 37°C for a total of 24 h and then centrifuged at 10 000 x *g* for 10 min. The pellet was resuspended in 150 µL of HBS before being deposited on a NS‐EM grid.

Measurements were performed at least in triplicate (indicated in each experiment), and data are represented as mean ± standard deviation (s.d.). The statistical analysis used is detailed for each experiment, using either a one‐way ANOVA with the Dunnett's multiple comparison test, or a Student's *t*‐test.

### 4.7 Negative‐Staining Electron Microscopy (NS‐EM)

NS‐EM carbon‐coated grids were prepared from protein samples incubated for 120 min at 37°C in HBS. A final concentration of 2 µm was used for this purpose as detailed in the Supporting Information, Experimental section. Measurements of fibril lengths were made manually with the ImageJ software [58].

### 4.8 Chemically‐Induced Dimerization of FKBP‐C^all^S

FKBP‐C^all^S sample was incubated in the absence or presence of AP20187 (Sigma–Aldrich, SML2838) as detailed in the Supporting Information, Experimental section. A part of the sample was analyzed by SEC, and excess AP20187 was removed using a PD Minitrap G‐25 column (Cytiva) equilibrated in HBS. Buffer‐exchanged samples were analyzed by turbidity and fluorimetry and NS‐EM in the same way as described above.

### 4.9 Multi‐Angle Laser‐Light Scattering (SEC‐MALLS)

The formation of FKBP‐C^all^S dimeric species upon addition of the AP20187 molecule was also assessed by SEC‐MALLS. For this purpose, the same protocol as previously described was followed, and the sample was centrifuged for 15 min at 10 000 x *g* at room temperature. 50 µL was injected at a flow rate of 0.6 mL/min on a Superdex S200 Increase 10/300 GL (Cytiva) column equilibrated in HBS controlled by an Ultimate 3000 HPLC (Thermo Scientific). Measurements were carried out on a Wyatt Dawn 8 angle MALLS equipped with an Optilab refractometer (Wyatt).

### 4.10 W^HeV^ C^all^S Variant Crosslinking by DTSSP

W^HeV^ C^all^S was cross‐linked in the presence of 2 mm DTSSP (Pierce, 21578). The entire protocol is described in the Supporting Information, Experimental section. The sample was analyzed by SEC and by turbidimetry and fluorimetry after a buffer‐exchange step in HBS, as described above, at a final concentration of 30 µm. NS‐EM grids were prepared after 2 h of incubation in HBS.

### 4.11 W^HeV^ and W^NiV^ Fibrils Preparation

500 µL of purified W^HeV^ and W^NiV^ were first incubated at a concentration of 114 and 84 µm, respectively, in HBS/6 m for 72 h. The solution was then desalted in HBS urea‐free buffer as described above and incubated for 72 h at room temperature at a final concentration of 57 µm (W^HeV^) and 42 µm (W^NiV^) in the presence of 100 µg/mL ampicillin. The solutions were then gently concentrated by centrifugation using an amicon device (100 kDa cut‐off, Cytiva). The retentate was then slowly centrifuged for 10 min at 1000× g to remove rare macroscopic aggregates before being aliquoted, flash‐frozen in liquid nitrogen, and stored at −80°C. In the case of W^HeV^, fibril enrichment was followed by NS‐EM prior to BLI experiments. To this end, the absorbance at 280 nm of both the W^HeV^ filtrate and retentate was measured, and 3.5 µL at 5 µm were deposited on an NS‐EM grid for each fraction.

### 4.12 Far‐UV Circular Dichroism (CD)

The foldedness of biotinylated STAT1_132‐682_ used for BLI experiments was assessed by CD spectroscopy. For this purpose, a sample of biotinylated STAT1_132‐682_ was first buffer‐exchanged in 10 mm sodium phosphate pH 7.4 using a PD SpinTrap G‐25 (Cytiva) and the far‐UV CD spectrum was then recorded in a 1‐mm thick quartz cuvette using a Jasco J‐810 spectropolarimeter flushed with 15 L/min gaseous N_2_. Spectra were recorded between 260 and 190 nm with a step of 0.5 nm at a speed of 100 nm/min, 4 s response time, 2 nm bandwidth. Ten acquisitions were recorded at a protein concentration of 3 µm, averaged and buffer‐subtracted. The same procedure was followed for the purified W^HeV^ and W^NiV^ fibrils that were analyzed at a concentration of 2.65 and 2.4 µm, respectively.

### 4.13 Biolayer Interferometry (BLI)

BLI was performed with an Octet R8 (Sartorius) thermostatted at 25°C. All the steps were performed in Dulbecco's PBS (Sigma, D8237) supplemented with 0.01% (*v/v*) Tween‐20 and under agitation (1000 rpm). STAT1_132‐682_ was first biotinylated using the EZ‐Link NHS‐PEG4‐Biotin kit (Thermo Scientific, A39259) according to the Sartorius guide (30 min, at room temperature) before being desalted using a PD SpinTrap G‐25 (Cytiva) equilibrated in HBS. Biotinylated STAT1_132‐682_ was then immobilized on streptavidin biosensors (Sartorius, 18–5019) at a concentration of 10 µg/mL for 60 s as described in Figure S9A. Kinetic analysis of the interaction between monomeric W^HeV^ and STAT1_132‐682_ was carried out using the following set of concentrations: 1.6, 0.8, 0.4, 0.2, and 0.1 µm. W^HeV^ fibrils concentration was 1.6 µm. Data corresponding to the reference sensor (empty sensor, highest concentration of analytes) were subtracted after checking the absence of non‐specific interaction (Figure S9A). Equilibrium dissociation constant (K_D_) and kinetic rate constants (ka, kd) were determined based on three different models as presented in Figure S9B,C. The best model fitting (2:1, heterogeneous ligand) was used for analysis.

### 4.14 Cellular Cultures

Human embryonic kidney 293T (HEK293T), A549, and Vero E6 cells were cultured in Dulbecco's Modified Eagle Medium (DMEM) (Gibco, Cat# 61965‐026) supplemented with 10% fetal bovine serum (FBS) (DMEM/10% FBS). The HEK293T cell line stably transduced with a firefly luciferase reporter under the control of a synthetic NF‐κB promoter (HEK293T NF‐κB_luc), and the HEK293T cell line expressing an ISRE‐dependent luciferase reporter (HEK293 ISRE_luc, kindly provided by the team of Pierre‐Olivier Vidalain) were cultured in DMEM/10% FBS, with 200 µg mL^−1^ hygromycine B (Invitrogen, Cat# 10687010) or 500 µg mL^−1^ G‐418 (MedChemTronica, Cat# HY‐17561), respectively. BEAS‐2B cells (kindly provided by the team of Pierre‐Olivier Vidalain) were cultured in DMEM/F‐12 (Gibco, Cat# 31331‐028) with 10% FBS. All the cells were cultured at 37°C in a 5% CO_2_ incubator.

### 4.15 Virus

WT HeV (Australia/1994/Horse18 isolate, GenBank accession number MN062017.1) was initially isolated from a horse and obtained from Porton Down Laboratory, UK [54, 59, 60]. Briefly, viral stock was produced by infecting Vero E6 cells at a multiplicity of infection of 0.001 in Opti‐MEM (Gibco, Cat# 51985042). After 1 h incubation at 37°C, the medium was replaced with DMEM with 5% FBS and cells were incubated at 37°C at 5% CO_2_ for 48 h. Viral supernatants were collected and centrifuged (400 g, 10 min, 4°C), aliquoted, and titrated in plaque‐forming units by classic dilution limit assay on Vero E6 cells.

### 4.16 Immunofluorescence

Transfected HEK293T cells were stained using a standard immunofluorescence protocol detailed in the Supporting Information, Experimental section, using a rabbit anti‐W^CTD^ antibody (GeneScript) (1:100) and Alexa Fluor 647 Donkey anti‐Rabbit IgG (H+L) Highly Cross‐Adsorbed Secondary antibody (Invitrogen, Cat# A‐31573) (1:500), before imaging on a Yokogawa HCS CQ1 spinning disk confocal system (PLATIM, SFR Biosciences Lyon).

### 4.17 Live Imaging and Quantification of W^HeV^ Condensates

HEK293T, BEAS‐2B and A549 cells were seeded in Ibidi µ‐Slide 8 Well and transfected 24 h later with 0.4 µg of the indicated constructs, using FuGENE 6 with a DNA:transfection reagent ratio of 1:4, or TransIT‐LT1 for A549 cells with a 1:3 ratio. They were stained at the indicated times post‐transfection using the TC‐ReAsH or FlAsH II In‐Cell Tetracysteine Tag Detection Kit and 1 µg/mL Hoechst 33342 when stated. To this end, cells were rinsed with Opti‐MEM, incubated for 45 min at room temperature with 2.5 µm of ReAsH/FlAsH‐EDT2 labeling reagents in Opti‐MEM, and finally rinsed with Opti‐MEM, allowing the visualization of *tc*‐tagged W^HeV^ on a Yokogawa HCS CQ1 confocal system. Z‐stacks of 10 to 15‐µm‐range with an imaging step of 0.7–1 µm were acquired in random areas of the wells. Maximum intensity projections were generated automatically by the microscope software. Quantification of the percentage of cells bearing condensates was performed with Fiji (v2.9.0). The total number of stained cells on the Z‐projections was counted semi‐automatically using Threshold, Fill Holes, Watershed, and Analyze Particles functions with constant parameters between projections (minimum size of 3000 px^2^). Cells with condensates were counted manually.

### 4.18 Time‐Lapse Imaging

HEK293T cells were seeded in Ibidi µ‐Slide 8 Well (175 000 cells/well) previously coated with 50 µg/mL poly‐D‐lysine. Cells were transfected 24 h later with 0.4 µg of the pCAGGS plasmid encoding tc‐W with FuGENE 6, with a DNA:transfection reagent ratio of 1:4. Different staining and imaging conditions using a Yokogawa HCS CQ1 confocal system (40x objective UPLSAPO40×2, Olympus) were tested. For Figure 6A,C, and Figure S7B, cells were rinsed 24 h later with Opti‐MEM, and 300 µL of Opti‐MEM with 1.5% FBS and 0.33 µm FlAsH was added. After 30 min of incubation at room temperature, cells were imaged every 50 min with a 488 nm laser (25% power) and 2.5 s exposition, at 37°C and with 5% CO_2_. For Figure 6B, cells were rinsed 24 h later with Opti‐MEM, and 300 µL of Opti‐MEM with 0.5% FBS and 0.67 µm FlAsH was added. After 30 min of incubation at room temperature, cells were imaged every 15 min with a 488 nm laser (1.5% power) and 3 s exposition, at 37°C and with 5% CO_2_. For Figure 7E, cells were rinsed 24 h later with Opti‐MEM, and 300 µL of Opti‐MEM with 2 µm FlAsH was added. After 30 min of incubation at room temperature, cells were imaged every 60 min with a 488 nm laser (7% power) and 2.5 s exposition, at 37°C and with 5% CO_2_. For Figure S7A, cells were rinsed 24 h later with Opti‐MEM, and 300 µL of Opti‐MEM with 1.5% FBS and 2 µm FlAsH were added. After 30 min of incubation at room temperature, cells were imaged every 60 min with a 488 nm laser (7% power) and 2.5 s exposition, at 37°C and with 5% CO_2_. For Figure S7C, cells were rinsed 24 h later with Opti‐MEM, and 300 µL of Opti‐MEM with 0.67 µm FlAsH were added. After 30 min of incubation at room temperature, cells were imaged every 15 min with a 488 nm laser (1.5% power) and 3 s exposition, at 37°C and with 5% CO_2_.

### 4.19 High‐Resolution Confocal Microscopy of Condensates

HEK293T cells were seeded in Ibidi µ‐Slide 8 Well and transfected as previously described with FuGENE 6. At 72 h post‐transfection, cells were rinsed with Opti‐MEM, incubated for 45 min at room temperature with 2.5 µm ReAsH in Opti‐MEM, and finally rinsed with Opti‐MEM. They were next imaged in Opti‐MEM on an abberior Instruments INFINITY Line confocal microscope with a 60x oil immersion objective (UPLXAPO60XO, NA 1.42, Olympus). To improve resolution, reduce background noise, and more globally increase the signal‐to‐noise ratio in order to be able to segment and quantify the condensates, the MATRIX detector was used for the acquisitions, requiring a pinhole size >10 and a pixel size of 65 nm. The following parameters were used for the acquisitions post‐treatment: Differential detection between 0.8 and 1.5 and Sharpening between 6 and 11. Analysis of the sphericity of the spots was performed with Fiji by cropping the stacks to keep only the individual spots; a Gaussian blur of 4 was then applied, images were thresholded, and the sphericity and volumes were finally measured with MorphoLibJ plugin [61] using the Analyze Regions 3D function.

### 4.20 3D Reconstruction and Quantification of Filamentous and Round‐Shaped Condensates

Z‐stack images acquired by confocal fluorescence microscopy in multiple channels were imported into Imaris software (Bitplane, version 10.0) and converted to the “.ims” format for subsequent analysis. Filamentous structures were reconstructed using the “Volume” creation tool within the 3D View module. For each fluorescence channel, the “Add New Surfaces” function was used to initiate object segmentation. The “Classify Surfaces” and “Object‐Object Statistics” features were enabled to allow detailed quantification of individual structures and their spatial relationships. To restrict the analysis to biologically relevant areas, the “Segment Only a Region of Interest” option was applied. Background fluorescence was reduced using the “Background Subtraction (Local Contrast)” algorithm. Surface boundaries were manually adjusted to accurately match the fluorescent signal observed in the original image slices. Additional filtering criteria, including “Quality,” “Mean Intensity,” “Sphericity,” and “Shortest Distance to Neighboring Object,” were applied to minimize artifacts or non‐specific signals. The same thresholding parameters were then uniformly applied across all images within each experimental group to ensure consistency. Round‐shaped condensates were analyzed following the same workflow, with the exception that filtering was limited to “Mean Intensity” and “Sphericity.” The “Smooth” option was enabled to refine surface contours, while the “Split Connected Items” option was disabled to preserve the continuity of individual filaments. After surface reconstruction, objects were categorized into subgroups based on parameters such as length, volume, and surface area. Quantitative data were exported for downstream statistical analysis. Subgroups were visually distinguished by adjusting surface color and transparency within the software.

### 4.21 Preparation of Samples for Electron Microscopy of Transfected Cells

HEK293T cells were seeded in 35‐mm gridded dishes (Ibidi, Cat# 81166) at a density of 600 000 cells per dish. Cells were transfected 24 h later as previously described using the TransIT‐LT1 reagent with 3.2 µg of plasmid DNA (empty pcDNA3.1(+) vector or tc‐tagged W construct). Live‐cell imaging was performed 24 and 48 h later using the TC‐ReAsH II In‐Cell Tetracysteine Tag Detection Kit on a Yokogawa HCS CQ1 confocal system to locate filament‐bearing cells. ReAsH‐EDT2 labeling reagent was used at a final concentration of 0.625 µm. Cells were imaged at 4 and 10× magnifications, and Z‐stacks of 8 to 12‐µm‐range with an imaging step of 0.8 µm were acquired with the 40× objective. Maximum intensity projections were generated automatically by the microscope software. After imaging, cells were fixed with 4% methanol‐free formaldehyde (Electron Microscopy Sciences, Cat# 15713) for 10 min, and kept in DPBS before tomography analysis.

### 4.22 Embedding and Ultramicrotomy of Transfected Cells, and EM Analyses

HEK293T cells were prepared following a regular EM sample preparation detailed in the Supporting Information, Experimental section. Briefly, cells were post‐fixed in 1% OsO_4_, *en bloc* contrasted in 1% uranyl acetate, dehydrated in ethanol, and embedded in Epon. The region of interest was recovered from the dish and targeted using Ibidi landmarks. One‐micron‐thick sections were collected on a glass slide. Sections of interest (those corresponding to the relevant z‐positions in the fluorescence stacks) were identified by toluidine blue staining and re‐embedded in pure Epon 812 and incubated at 60°C for approximately 12 h. The samples were then immersed in liquid nitrogen to remove the resin‐embedded sections from the slide (cracking). 70–90 nm ultrathin sections were post‐stained with uranyl acetate and lead citrate. Images were taken using a Tecnai T12 Spirit 120 kV.

### 4.23 Assessment of Cell Death by Fluorescence Microscopy

HEK293T cells were seeded in Ibidi µ‐Slide 8 Well (150 000 cells/well) previously coated with 50 µg mL^−1^ poly‐D‐lysine. Cells were transfected 24 h later with 0.4 µg of the indicated plasmids with FuGENE 6, with a DNA:transfection reagent ratio of 1:4. The next day, activated Caspase‐1 was stained using the Caspase 1 Staining Kit (Far Red) (Abcam, Cat ab270784). The staining solution was prepared according to the manufacturer's instructions, and after removing the medium, 100 µL were added per well. Cells were incubated at 37°C for 45 min, then the staining solution was removed, and the cells were rinsed twice with Opti‐MEM. Cells were next stained with a mix of 2.5 µm FlAsH from the TC‐FlAsH II In‐Cell Tetracysteine Tag Detection Kit (Invitrogen, Cat T34561), 3 µg/mL propidium iodide, and 1 µg mL^−1^ Hoechst in Opti‐MEM, at room temperature for 45 min in the dark. Cells were rinsed with Opti‐MEM and imaged with a Yokogawa HCS CQ1 confocal system in Opti‐MEM. Positive control cells were incubated with 0.01% Triton X‐100 in Opti‐MEM for 10 min prior to staining.

### 4.24 Cell Viability/Metabolic Assay

HEK293T cells were seeded in poly‐D‐lysine‐coated 96‐well black plate (Corning, 354640) and transfected 24 h later with 0.2 µg of the indicated plasmids with the TransIT‐LT1 reagent. Cell viability was measured the next day using alamarBlue Cell Viability Reagent (Invitrogen, DAL1025) by quantifying fluorescence intensity on a Tristar 5 microplate reader, according to the manufacturer's instructions, using an excitation wavelength of 570 ± 7.5 nm, an emission wavelength of 585 ± 7.5 nm, and an exposure time of 100 ms.

### 4.25 Luciferase Assay

HEK293T NF‐κB_luc and HEK293 ISRE_luc cells were seeded in 96‐well plates (50 000 cells/well) previously coated with 50 µg mL^−1^ poly‐D‐lysine, and transfected 24 h later with 0.2 µg of an empty pCAGGS vector or pCAGGS plasmids coding for *tc*‐tagged W^HeV^ variants, using FuGENE 6 (Promega, Cat# E2691) according to the manufacturer's instructions, with a DNA:reagent ratio of 1:4. The medium was replaced 24 h later with DMEM/10% FBS supplemented or not with 50 ng mL^−1^ human IL‐1β (PeproTech, Cat 200–01B) or 100 U mL^−1^ universal type I IFN (PBL Assay Science, Cat 11200–1). Luciferase activity was measured after 5 h of incubation at 37°C using the Bright‐Glo Luciferase Assay System (Promega, E2620). The medium was replaced with 50 µL per well of DMEM/10% FBS, and 50 µL per well of Bright‐Glo Luciferase Assay Substrate reconstituted in assay buffer was added before quantifying luminescence in a 96‐well white opaque plate (1 s per well) on a Tristar 5 microplate reader (Berthold Technologies GmbH & co.KG)

### 4.26 Intracellular ROS Quantification

Intracellular ROS were quantified in HEK293T cells seeded and transfected as described for immunofluorescence staining, using the Fluorometric Intracellular ROS Kit (Sigma–Aldrich, MAK145). Briefly, medium was replaced with DMEM/10% FBS and the ROS detection master mix was then added with a medium:master mix ratio of 0.9:1. Cells were incubated for 1 h in a 5% CO2, 37°C incubator, and then imaged on a Yokogawa HCS CQ1 confocal microscope. For each condition, single optical sections from nine random areas in the wells were acquired. Images were then analyzed with Fiji (v2.9.0), by first subtracting the background (rolling ball radius of 50 pixels) and then measuring the mean gray value for each field of view. For ROS detection in infected cells, HEK293T cells seeded in Ibidi µ‐Slide 8 Well were infected with 100 PFU of HeV in Opti‐MEM or left uninfected. After 3 h of incubation at 37°C, the medium was replaced with DMEM/10% FBS, and ROS were quantified 24 h later using the Fluorometric Intracellular ROS Kit. Cells were imaged with a Leica DMIRB epifluorescence microscope with a 10x objective and analyzed with Fiji.

### 4.27 Western Blot

HEK293T cells were seeded in 12‐well plates (700 000 cells/well) and transfected 24 h later with 1.4 µg of the indicated constructs using FuGENE 6 as previously described. The next day, cells were rinsed with ice‐cold DPBS, collected on ice in RIPA buffer (Invitrogen, Cat 89901) supplemented with Halt Protease and Phosphatase Inhibitor Cocktail (Invitrogen, Cat 78442), and sonicated using a Bioruptor Plus (Hologic Diagenode). Protein samples were then prepared for gel loading in NuPAGE LDS Sample Buffer (Invitrogen, Cat NP0008) and NuPAGE Sample Reducing Agent (Invitrogen, NP0009), heated at 96°C for 10 min, and separated by electrophoresis using 10% Mini‐PROTEAN TGX Precast Protein Gels (Bio‐Rad, Cat 4561036) in TG‐SDS buffer (Euromedex, Cat EU0510). Separated proteins were then transferred on PVDF membranes using Trans‐Blot Turbo Midi 0.2 µm PVDF Transfer Packs (Bio‐Rad, Cat 1704157) and the Trans‐Blot Turbo Transfer System (Bio‐Rad, Cat 1704150). The membranes were then blocked with TBS buffer + 0.05% Tween 20 (TBST) + 5% milk for 1 h under agitation, rinsed with TBST and incubated with a rabbit anti‐W^CTD^ antibody (GeneScript) or a mouse anti‐GAPDH antibody (Sigma–Aldrich, Cat MAB374) diluted in TBST + 0.2% milk (both 1/1000), for 1 h at room temperature or overnight at 4°C, respectively. They were rinsed with TBST three times for 10 min, and incubated with Anti‐Rabbit IgG (H+L), HRP Conjugate (Promega, Cat W4011) or Anti‐Mouse IgG (H+L), HRP Conjugate (Promega, cat W4021) secondary antibodies diluted in TBST + 5% milk (both 1/10 000). After three rinses with TBST for 10 min, revelation was performed using the SuperSignal West Pico PLUS Chemiluminescent Substrate (Thermo Scientific, Cat 34580) on a ChemiDoc (Bio‐Rad).

### 4.28 Colocalization Experiments Between STAT1 and W

HEK293T cells were seeded in Ibidi µ‐Slide 8 Well (Ibidi, Cat# 80826) previously coated with 50 µg/mL poly‐D‐lysine. Cells were transfected 24 h later at ∼70% confluency with 0.3 µg indicated tc‐tagged W^HeV^ variant construct and 0.2 µg STAT1‐GFP expressing plasmid (a kind gift from Viktor Volchkov), using FuGENE 6 (Promega, Cat# E2691) with a DNA:transfection reagent ratio of 1:4. Cells were stained at 24 h post‐transfection using TC‐ReAsH II In‐Cell Tetracysteine Tag Detection Kit (Invitrogen, Cat#T34562). Briefly, cells were rinsed with Opti‐MEM, incubated 45 min at room temperature protected from light with 2.5 µm of ReAsH labeling reagent diluted in Opti‐MEM, and finally rinsed with Opti‐MEM. Cells were fixed in 4% paraformaldehyde (PFA) for 20 min, washed with PBS and mounted with Ibidi Mounting Medium (Ibidi, Cat# 50001). Imaging was performed using a Zeiss LSM 980AS2 confocal microscope (PLATIM, SFR Biosciences Lyon).

### 4.29 Statistical Analysis

Statistical analysis for each in vitro experiment is detailed in the corresponding figure legends. Figures 7, 8, 9, 10: all data are mean ± SD. Figure 7C: *n* = 3 cells. Figure 7D: *n* = 5 spots. Figure 8B: *n* = 6. Data normalized relative to EV condition (set at 100%) and analyzed by one‐way ANOVA completed by a Tukey's multiple comparison test (two‐sided, alpha value = 0.05). ^***^ *p* <0.001. Figure 9C: *n* = 7. Data analyzed by two‐way ANOVA followed by a Tukey's multiple comparisons test (two‐sided, alpha value = 0.05). ^**^ *p* <0.01, ^***^ *p* <0.001, ^****^ *p* <0.0001. Figure 10B: *n* = 9. Data normalized relative to EV condition (set at 100%) and analyzed by Kruskal–Wallis test completed by a Dunn's multiple comparison test (two‐sided, alpha value = 0.05). ^**^ *p* < 0.01. Figure 10C: Data analyzed by Mann–Whitney test (two‐sided, alpha value = 0.05). ^*^ *p* < 0.05. Figure 10D,E: *n* = 5 (D), 6 (E). Data normalized relative to EV non‐stimulated condition (set at 100%) and analyzed by unpaired *t*‐tests (two‐sided, alpha value = 0.05). ^*^ *p* <0.05, ^**^ *p* <0.01, ^***^ *p* <0.001, ^****^ *p* <0.0001. All data were analyzed with GraphPad Prism 9.

## Author Contributions

F.G., A.L., C.M. and S.L. designed the experiments; F.G. performed all the in vitro experiments except for the mass spectrometry analyses that were performed by Patrick Fourquet, mass spectrometry facility of Marseille Proteomics; A.L. performed transfections, microscopy studies and relative quantitative analyses, ROS and cytotoxicity quantification, infections in the BSL4 and analysis of impact on innate immunity pathways; Ca.M. contributed to the cloning of some pCAGGS constructs, transfections and microscopy studies on transfected cells, and quantifications, under the supervision of A.L.; C.B. and F.G. generated all the constructs except for the constructs of the W^HeV^ proteins with an N‐terminal tc tag and the tc‐mStayGold that were generated by A.L. and Ca.M.; D.P. provided guidance on NS‐EM studies; G.P. carried out the ultramicrotomy experiments and related EM analyses; A.B. performed the STAT1‐W colocalization studies; D.G. provided guidance on transfection and confocal microscopy studies and suggested to perform experiments with FKBP; P.Y.L provided guidance on the use of the tc tag and supervised Y.G. in the computer‐based image analysis; Y.G. performed the Imaris 3D reconstructions of nuclear aggregates; E.O. performed the Western blot analysis of transfected cells; F.G, A.L., C.M., A.B. and S.L analyzed the data; F.G. and S.L. wrote the first draft of the manuscript; F.G. generated Figures 1, 2, 3 and Figure 12 A.B. generated Figure 11; A.L. generated Figure 4, 5, 6, 7, 8, 9, 10; F.G., A.L., C.M. and S.L generated the final version of the manuscript, and all the authors reviewed and edited it.

## Funding

This work was carried out with the financial support of the Agence Nationale de la Recherche (ANR), specific project Heniphase (ANR‐21‐CE11‐0012‐01) that was labeled by the Eurobiomed and Lyon Biopole competitiveness clusters. It was also partly supported by the CNRS and by the French Infrastructure for Integrated Structural Biology (FRISBI) (ANR‐10‐INSB‐0005). This work was also supported by the EquipEx+ Spatial‐Cell‐ID under the “Investissements d'avenir” program (ANR‐21‐ESRE‐00016). F.G. was supported by a post‐doctoral fellowship from the FRM (Fondation pour la Recherche Médicale). G.P. was supported by a joint doctoral fellowship from the AID (Agence Innovation Défense) and Aix‐Marseille University (AMU). A.B. was supported by the French gouvernement through the France 2030 program and the ANRS | MIE, specific project ANRS‐23‐PEPR‐MIE 0008. The funders had no role in the design of the study; in the collection, analyses, or interpretation of data; in the writing of the manuscript; or in the decision to publish the results. A CC‐BY public copyright license has been applied by the authors to the present document and will be applied to all subsequent versions up to the author accepted manuscript arising from this submission, in accordance with the grant's open access conditions.

## Conflicts of Interest

The authors declare no conflicts of interest.

## Supporting information




**Supporting File 1**: advs76272‐sup‐0001‐SuppMat.docx.

**Supporting File 2**: advs76272‐sup‐0002‐MovieS1.mp4.

**Supporting File 3**: advs76272‐sup‐0003‐MovieS2.mp4.

## Data Availability

Following publication, the data presented in the current study will be deposited within the French governmental repository Dat'AMU (https://entrepot.recherche.data.gouv.fr/dataverse/univ‐amu).
